# Association of plasma biomarkers of fruit and vegetable intake with incident type 2 diabetes: EPIC-InterAct case-cohort study in eight European countries

**DOI:** 10.1136/bmj.m2194

**Published:** 2020-07-08

**Authors:** Ju-Sheng Zheng, Stephen J Sharp, Fumiaki Imamura, Rajiv Chowdhury, Thomas E Gundersen, Marinka Steur, Ivonne Sluijs, Yvonne T van der Schouw, Antonio Agudo, Dagfinn Aune, Aurelio Barricarte, Heiner Boeing, María-Dolores Chirlaque, Miren Dorronsoro, Heinz Freisling, Douae El-Fatouhi, Paul W Franks, Guy Fagherazzi, Sara Grioni, Marc J Gunter, Cecilie Kyrø, Verena Katzke, Tilman Kühn, Kay-Tee Khaw, Nasser Laouali, Giovanna Masala, Peter M Nilsson, Kim Overvad, Salvatore Panico, Keren Papier, J Ramón Quirós, Olov Rolandsson, Daniel Redondo-Sánchez, Fulvio Ricceri, Matthias B Schulze, Annemieke M W Spijkerman, Anne Tjønneland, Tammy Y N Tong, Rosario Tumino, Elisabete Weiderpass, John Danesh, Adam S Butterworth, Elio Riboli, Nita G Forouhi, Nicholas J Wareham

**Affiliations:** 1 MRC Epidemiology Unit, University of Cambridge School of Clinical Medicine, Institute of Metabolic Science, Cambridge Biomedical Campus, Cambridge CB2 0QQ, UK; 2 Key Laboratory of Growth Regulation and Translation Research of Zhejiang Province, School of Life Sciences, Westlake University, Hangzhou, China; 3 MRC/BHF Cardiovascular Epidemiology Unit, Department of Public Health and Primary Care, University of Cambridge, Cambridge, UK; 4 Vitas AS, Oslo, Norway; 5 Julius Centre for Health Sciences and Primary Care, University Medical Center Utrecht, Utrecht University, Utrecht, Netherlands; 6 Unit of Nutrition and Cancer, Cancer Epidemiology Research Programme, Catalan Institute of Oncology, Group of Research on Nutrition and Cancer, Bellvitge Biomedical Research Institute, L'Hospitalet of Llobregat, Barcelona, Spain; 7 School of Public Health, Imperial College London, London, UK; 8 Department of Nutrition, Bjørknes University College, Oslo, Norway; 9 Department of Endocrinology, Morbid Obesity and Preventive Medicine, Oslo University Hospital, Oslo, Norway; 10 Navarra Public Health Institute, Pamplona, Spain; 11 Department of Epidemiology, German Institute of Human Nutrition Potsdam-Rehbruecke, Nuthetal, Germany; 12 Department of Epidemiology, Regional Health Council, IMIB-Arrixaca, Murcia University, Murcia, Spain; 13 CIBER in Epidemiology and Public Health (CIBERESP), Madrid, Spain; 14 Public Health Division of Gipuzkoa, San Sebastian, Spain; 15 International Agency for Research on Cancer, Lyon, France; 16 Centre of Research in Epidemiology and Population Health, UMR 1018 Inserm, Institut Gustave Roussy, Paris-Sud Paris-Saclay University, Villejuif, France; 17 Department of Clinical Sciences, Lund University, Malmö, Sweden; 18 Luxembourg Institute of Health (LIH), Department of Population Health, Strassen, Luxembourg; 19 Epidemiology and Prevention Unit, Milan, Italy; 20 Danish Cancer Society Research Centre, Copenhagen, Denmark; 21 Division of Cancer Epidemiology, German Cancer Research Centre, Heidelberg, Germany; 22 Department of Public Health and Primary Care, University of Cambridge, Cambridge, UK; 23 Cancer Risk Factors and Life-Style Epidemiology Unit, Institute for Cancer Research, Prevention and Clinical Network, Florence, Italy; 24 Department of Public Health, Aarhus University, Aarhus, Denmark; 25 Department of Cardiology, Aalborg University Hospital, Aarhus, Denmark; 26 Department of Clinical Medicine and Surgery, Federico II University, Naples, Italy; 27 Cancer Epidemiology Unit, Nuffield Department of Population Health, University of Oxford, Oxford, UK; 28 Public Health Directorate, Asturias, Spain; 29 Department of Public Health and Clinical Medicine, Family Medicine, Umeå University, Umeå, Sweden; 30 Andalusian School of Public Health, Granada, Spain; 31 Institute of Biosanitary Research of Granada, Granada, Spain; 32 Department of Clinical and Biological Sciences, University of Turin, Turin, Italy; 33 Unit of Epidemiology, Regional Health Service ASL TO3, Grugliasco, Turin, Italy; 34 Department of Molecular Epidemiology, German Institute of Human Nutrition Potsdam-Rehbruecke, Nuthetal, Germany; 35 German Centre for Diabetes Research, Neuherberg, Germany; 36 University of Potsdam, Institute of Nutritional Sciences, Potsdam, Germany; 37 National Institute for Public Health and the Environment, Bilthoven, Netherlands; 38 Cancer Registry and Histopathology Department, Azienda Sanitaria Provinciale, Ragusa, Italy; 39 NIHR Blood and Transplant Research Unit in Donor Health and Genomics, Department of Public Health and Primary Care, University of Cambridge, Cambridge, UK; 40 British Heart Foundation Cambridge Centre of Excellence, Division of Cardiovascular Medicine, Addenbrooke's Hospital, Cambridge, UK; 41 Department of Human Genetics, Wellcome Trust Sanger Institute, Hinxton, Cambridge, UK

## Abstract

**Objective:**

To investigate the association of plasma vitamin C and carotenoids, as indicators of fruit and vegetable intake, with the risk of type 2 diabetes.

**Design:**

Prospective case-cohort study.

**Setting:**

Populations from eight European countries.

**Participants:**

9754 participants with incident type 2 diabetes, and a subcohort of 13 662 individuals from the European Prospective Investigation into Cancer and Nutrition (EPIC) cohort of 340 234 participants: EPIC-InterAct case-cohort study.

**Main outcome measure:**

Incident type 2 diabetes.

**Results:**

In a multivariable adjusted model, higher plasma vitamin C was associated with a lower risk of developing type 2 diabetes (hazard ratio per standard deviation 0.82, 95% confidence interval 0.76 to 0.89). A similar inverse association was shown for total carotenoids (hazard ratio per standard deviation 0.75, 0.68 to 0.82). A composite biomarker score (split into five equal groups), comprising vitamin C and individual carotenoids, was inversely associated with type 2 diabetes with hazard ratios 0.77, 0.66, 0.59, and 0.50 for groups 2-5 compared with group 1 (the lowest group). Self-reported median fruit and vegetable intake was 274 g/day, 396 g/day, and 508 g/day for participants in categories defined by groups 1, 3, and 5 of the composite biomarker score, respectively. One standard deviation difference in the composite biomarker score, equivalent to a 66 (95% confidence interval 61 to 71) g/day difference in total fruit and vegetable intake, was associated with a hazard ratio of 0.75 (0.67 to 0.83). This would be equivalent to an absolute risk reduction of 0.95 per 1000 person years of follow up if achieved across an entire population with the characteristics of the eight European countries included in this analysis.

**Conclusions:**

These findings indicate an inverse association between plasma vitamin C, carotenoids, and their composite biomarker score, and incident type 2 diabetes in different European countries. These biomarkers are objective indicators of fruit and vegetable consumption, and suggest that diets rich in even modestly higher fruit and vegetable consumption could help to prevent development of type 2 diabetes.

## Introduction

The global burden of type 2 diabetes has risen over the past decades and its prevention is a public health priority.^1^ High fruit and vegetable intake has been suggested to have an important role in prevention of this disorder.^2^ Evidence from prospective studies linking fruit and vegetable intake with type 2 diabetes is inconsistent and weak,^3^ ^4^ ^5^, and evidence from randomised controlled trials is sparse.^6^ Previous research studies have typically used dietary food frequency questionnaires to assess fruit and vegetable intake, which are subject to measurement error and recall bias.^7^ Circulating plasma vitamin C and carotenoids have been proposed as objective biomarkers of fruit and vegetable intake, with evidence for their validity from observational and experimental studies.^8^ ^9^ ^10^ ^11^ ^12^ In meta-analyses of randomised controlled trials, groups provided with more fruits and vegetables had increased blood concentrations of a panel of fruit and vegetable related biomarkers.^10^ ^11^ ^12^ A meta-analysis of up to 96 intervention studies found that blood vitamin C and several carotenoids were the most consistently responsive biomarkers for fruit and vegetable intake.^11^ In individual participant meta-analysis of controlled intervention studies, evidence was found of a positive dose-response association between fruit and vegetable consumption and biomarker concentrations.^12^ Moreover, in comparative analyses, compliance at group level with fruit and vegetable interventions was indicated equally well by blood vitamin C or individual carotenoids.^10^ Thus, investigation of the association between these biomarkers and type 2 diabetes could provide insight into the association of fruit and vegetable intake with this disorder.

Results from previous small studies describing the association between circulating plasma carotenoids and the incidence of type 2 diabetes are inconclusive, with an inverse association being reported in some studies^9^ ^13^ ^14^ ^15^ and others finding no significant association.^16^ Only one previously published prospective cohort study has examined circulating vitamin C and incident type 2 diabetes^17^ reporting an inverse association between the concentration of plasma vitamin C and incident type 2 diabetes in a population in the United Kingdom, but evidence is lacking in other countries or populations with different lifestyles and dietary behaviours.

Our study aimed at examining the association of baseline levels of circulating vitamin C and carotenoids with incident type 2 diabetes in the European Prospective Investigation into Cancer and Nutrition (EPIC)-InterAct study, which is based on more than 340 000 community based adults from eight European countries. We also aimed to construct a composite biomarker score to examine the association of the combination of biomarkers with incident type 2 diabetes.

## Methods

### Study design and population

The EPIC-InterAct study is a prospective case-cohort study, nested within the European EPIC study.^18^ In brief, cases of incident type 2 diabetes occurring in eight of the 10 EPIC countries (Denmark, France, Germany, Italy, Netherlands, Spain, Sweden, and UK) between 1991 and 2007 were ascertained and verified by the InterAct consortium across 26 study centres. We ascertained and verified 12 403 individuals with incident type 2 diabetes over 3.99 million person years of follow-up from a cohort of 340 234 participants with stored blood and buffy coat. From this cohort, a centre stratified subcohort was assembled by randomly selecting 16 835 individuals. A total of 16 154 participants remained in the subcohort after exclusion of 548 with prevalent diabetes, 129 with uncertain diabetes status, and 4 with diabetes after censoring. The case-cohort design has the advantages of temporal sequence and power of a cohort study (in that it involves the complete number of incident cases) with the measurement efficiency of a case-control study. The random subcohort included 778 participants with verified incident type 2 diabetes, which is a design feature of the case-cohort approach.^18^ We further excluded 6543 participants with no plasma samples available for the measurement of plasma vitamin C (including all Swedish samples (n=5401)) and 4952 with no carotenoids (including one Swedish centre, Malmo (n=3556)). We subsequently excluded those with samples of low volume or samples that failed in biochemical analysis or were haemolysed (n=218 for vitamin C; n=85 for carotenoids). We, therefore, included a total of 22 833 participants, with 9754 participants with incident type 2 diabetes and 13 662 subcohort participants, with an average follow up of 9.7 years (supplemental figure S1). As a design feature of the case-cohort study the final eligible subcohort included 583 participants with incident type 2 diabetes. For the analysis of different biomarkers, the sample size varied according to the number of missing data for the biomarkers or covariates, and the final sample size for each biomarker analysis is presented.

All participants gave written informed consent and the study was approved by the local ethics committee in the participating centres, and the internal review board of the International Agency for Research on Cancer.

### Ascertainment and verification of cases of type 2 diabetes

Ascertainment of cases of incident type 2 diabetes up until 31 December 2007 involved a review of self-reported data, linkage to primary and secondary care registers, use of medication (drug registers), hospital admissions, and mortality data, which we have described in detail previously.^18^ No cases were ascertained solely by self-report because we confirmed any self-reported case with at least one other independent source of information. Cases of type 2 diabetes in Denmark and Sweden were not ascertained by self-report but were identified through local and national diabetes and pharmaceutical registers, and were considered verified.

### Measurement of plasma vitamin C and carotenoids

Non-fasting blood samples were collected at the baseline visit of the EPIC study. Plasma samples stored at −196°C (or at −150°C for samples from Denmark) were shipped to Vitas Analytical Services (Oslo, Norway) for the measurement of plasma vitamin C and six individual carotenoids (α carotene, β carotene, lycopene, lutein, zeaxanthin, β cryptoxanthin) by high performance liquid chromatography-ultraviolet methods, as described previously, by staff masked to participant information.^19^ ^20^ Assays for plasma vitamin C and carotenoids were performed on separate plasma aliquots, with treatment methods and chromatographic procedures.^19^ ^20^ For the vitamin C assay, meta-phosphoric acid was added to stabilise the vitamin C and prevent oxidation. For carotenoids, internal standard β apo-8-carotenal and butylated hydroxytoluene (as an antioxidant) were added before treatment. Coefficients of variation were 4.2-4.5% for vitamin C and 2.7-6.7% for carotenoids. The lower limit of detection was 0.7 µmol/L for vitamin C, 0.009 µmol/L for α carotene and β carotene, 0.018 µmol/L for lycopene, 0.009 µmol/L for lutein and zeaxanthin, and 0.007 µmol/L for β cryptoxanthin. We imputed random values between zero and the lower limit of detection for those below the lower limit (1.0% for vitamin C, 1.7% for α carotene, 6.7% for zeaxanthin, and <0.5% for others).

To assess the reproducibility of the assay of vitamin C and carotenoids after long term storage, we compared these biomarkers in the period of measurement for this study (2013-14) with those previously measured in 2004^9^ ^17^ in a subset of Norfolk (UK) participants of the EPIC study (n=1582 for vitamin C; n between 520 and 678 for individual carotenoids). The reproducibility for the measurement of these biomarkers was high for most biomarkers (r=0.89-0.93), and moderate for zeaxanthin (r=0.63).

### Measurement of other baseline characteristics

Baseline weight, height, and waist circumference were collected by trained health professionals during a visit to a study centre, except in France and in the Oxford cohort.^21^ Baseline dietary information was collected using a dietary questionnaire that was self-administered or administered by an interviewer, which was developed within each country to estimate the usual food intake of participants.^21^ ^22^ ^23^ Information on baseline physical activity, smoking status, sociodemographic factors, and medical history was obtained from self-administered questionnaires.^21^

Serum lipid biomarkers (except for Umeå centre in Sweden, where plasma was used), including total cholesterol, high density lipoprotein cholesterol, and triglycerides were measured at SHL (Stichting Ingenhousz Laboratory, Etten-Leur, Netherlands). Low density lipoprotein cholesterol was calculated based on the Friedewald formula.^24^ Both high and low density lipoprotein cholesterol were considered as confounders because both can influence carotenoid levels (as fat soluble vitamins) and could influence the association between nutritional biomarker and disease.^9^ ^25^

### Statistical analysis

All the statistical analyses were performed in Stata 14 (Statacorp, College Station, TX). The concentration of total carotenoids was calculated as the sum of the six individual carotenoids. Plasma vitamin C, total, and six individual carotenoids (α carotene, β carotene, lycopene, lutein, zeaxanthin, β cryptoxanthin) were the exposures for this analysis. A composite biomarker score was generated by calculating the average of the standardised values (each value was standardised using the mean and standard deviation from the subcohort) of vitamin C and six individual carotenoids, as previously described.^9^ The Spearman correlations between plasma vitamin C and six individual carotenoids were calculated in the subcohort. In cross sectional analyses, to identify potential correlates of plasma vitamin C and carotenoids, we log transformed the values of the total and individual carotenoids, owing to their skewed distributions. In the subcohort, we used linear regression to estimate country-specific β coefficients and 95% confidence intervals, representing the associations of demographic, lifestyle, and dietary factors with plasma vitamin C, total and six individual carotenoids, and the composite biomarker score, with mutual adjustment for all the demographic, lifestyle, and dietary factors in the model. We then pooled the estimated associations across countries using random effects meta-analysis.

To help interpret the results of the composite biomarker score in relation to fruit and vegetable intake, we calculated the difference in total fruit and vegetable intake for each one standard deviation higher composite biomarker score in the subcohort using a linear regression. These analyses included adjustment for age, sex, centre, physical activity, smoking status, employment, marital status, education, alcohol intake, total energy intake, body mass index, waist circumference, and dietary covariates (intake of potatoes, cereal and cereal products, milk and dairy products, fruit and vegetable juice, soft drinks, fish, red meat, legumes, egg and egg products, nuts and seeds, offal, and vitamin supplement use). In addition, we used a regression model with restricted cubic splines (three knots) to assess the shape of the association of the composite biomarker score with fruit and vegetable intake in the subcohort, adjusted for the same covariates as above.

We used Prentice weighted Cox regression^26^ to estimate country-specific hazard ratio and 95% confidence interval for incident type 2 diabetes, comparing biomarkers divided into five equal groups (from the lowest, group 1 to the highest, group 5) and for each one standard deviation (groups 1-5 and the standard deviation were both calculated in the subcohort). We then pooled the estimates using random effects meta-analysis. We fitted three statistical models, adjusting for potential sociodemographic, lifestyle behavioural, biochemical, and anthropometric confounders: model 1a adjusted for age (as underlying time scale, continuous), sex, and centre; model 1b, as model 1a, plus physical activity (inactive, moderately inactive, moderately active, active), smoking status (never, former, current), employment (no, yes), marital status (single, married, separated/divorced, widowed), education (low, middle, high), alcohol drinking (never, 0 to <6, 6 to <12, 12 to <24, and ≥24 g/day), total energy intake (continuous), high density lipoprotein cholesterol (continuous; only for carotenoids analyses) and low density lipoprotein cholesterol (continuous; only for carotenoids analyses); and model 2, as model 1b, plus body mass index (continuous) and waist circumference (continuous). Based on model 2, we further modelled restricted cubic spline terms (three knots) for analysis of each biomarker to assess the shape of their associations with incident type 2 diabetes.^27^ ^28^ To investigate the association with type 2 diabetes of an estimated daily fruit and vegetable intake that meets or exceeds the “five-a-day” recommendation (≥400 g/day) compared with not meeting the recommendation (<400 g/day), we categorised participants into two groups: biomarker predicted five or more portions a day and fewer than five portions a day, using the value of the composite biomarker score, which equated to 400 grams a day of fruit and vegetable intake in the prediction model, as the cut-off point.

In sensitivity analyses, we imputed missing values of covariates using multiple imputation with chained equations, 29 to assess the effect of missing data on the biomarker-disease associations; we performed analysis in each of 10 imputed datasets and combined the estimates using Rubin’s rules. The imputation model included all covariates from the analysis model, the event variable, and the Nelson-Aalen estimate of cumulative hazard.^29^ To examine the influence of each individual biomarker on the composite biomarker score results, we amended the composite biomarker score by excluding one biomarker at a time, and then examined its association with type 2 diabetes. We also assessed robustness of the results from model 2 to take account****of other potential confounders, including mutual adjustment for the other individual nutritional biomarkers (vitamin C and six individual carotenoids), use of vitamin supplements, season of blood draw, family history of diabetes, baseline prevalence of stroke, coronary heart diseases or cancers (excluding non-melanoma skin cancer), hormone use and menopausal status in women, genetic risk score for body mass index and for insulin resistance, and a diet quality score (Mediterranean diet score). The genetic risk scores for body mass index and insulin resistance were both unweighted, and were calculated as the sum of the number of risk alleles for body mass index (97 genetic variants)^30^ or insulin resistance (53 genetic variants).^31^ We also accounted for potential reverse causality by excluding people with haemoglobin A1c greater than or equal to 6.5% (48 mmol/mol) at baseline or those confirmed as having type 2 diabetes within the first two years or first four years after baseline. We performed further sensitivity analysis by excluding participants with baseline cancer or cardiovascular disease or by restricting the analysis to the first eight years of follow-up. We tested for multiplicative interaction between each nutritional biomarker and the following prespecified variables: age, sex, body mass index, physical activity, smoking status, season of blood draw, and vitamin supplement use. We used a lower threshold for significance based on the Bonferroni correction. Analyses within subgroups defined by the variables were performed if the P value for interaction was <0.05.

### Patient and public involvement

No patients were involved in setting the research question, nor were they involved in the design and implementation of the study. No plans exist to involve patients in dissemination of the results.

## Results

### Population characteristics

Baseline mean (standard deviation) concentration of plasma vitamin C and total carotenoids was 36.3 (18.3) and 1.3 (0.7) µmol/L, respectively, among individuals who developed type 2 diabetes compared with 42.3 (19.2) µmol/L and 1.7 (0.8) µmol/L in subcohort participants (table 1). In the whole subcohort, participants in Germany had the highest mean levels of plasma vitamin C (48.4 (standard deviation 19.2) µmol/L), whereas those from Italy had the lowest levels (36.6 (18.6) µmol/L). Participants from France had the highest mean plasma total carotenoid levels (2.3 (1.0) µmol/L), whereas those from Denmark had the lowest levels (1.3 (0.8) µmol/L; table 1). Plasma vitamin C, total and individual carotenoids were all positively correlated with each other (supplemental table S1).

**Table 1:** Distribution of plasma vitamin C and carotenoids by case status and by population characteristics in the subcohort of EPIC-InterAct study

Subgroup	Vitamin C (µmol/L)		Total carotenoids (µmol/L)		α carotene (µmol/L; median (IQR))	β carotene (µmol/L; median (IQR))	Lycopene (µmol/L; median (IQR))	Lutein (µmol/L; median (IQR))	Zeaxanthin (µmol/L; median (IQR))	β cryptoxanthin (µmol/L; median (IQR))	Composite biomarker score (median (IQR))
	No	Mean (SD)	No	Median (IQR)							
Case status:										
Non-cases	12 034	42.6 (19.2)	13 039	1.54 (1.11-2.08)	0.07 (0.04-0.14)	0.38 (0.24-0.59)	0.42 (0.26-0.61)	0.26 (0.19-0.36)	0.04 (0.02-0.07)	0.20 (0.11-0.37)	−0.04 (−0.37-0.33)
Cases of type 2 diabetes	8984	36.3 (18.3)	9703	1.19 (0.84-1.68)	0.05 (0.03-0.09)	0.26 (0.16-0.41)	0.34 (0.20-0.53)	0.22 (0.15-0.31)	0.03 (0.02-0.06)	0.15 (0.08-0.29)	−0.30 (−0.60-0.05)
Total subcohort*	12 589	42.3 (19.2)	13 618	1.52 (1.09-2.07)	0.07 (0.04-0.13)	0.37 (0.23-0.58)	0.41 (0.25-0.61)	0.26 (0.18-0.36)	0.04 (0.02-0.07)	0.20 (0.11-0.37)	−0.05 (−0.38-0.32)
Country:										
France	512	45.0 (17.1)	536	2.22 (1.63-2.83)	0.18 (0.12-0.29)	0.78 (0.52-1.08)	0.42 (0.27-0.59)	0.36 (0.27-0.49)	0.04 (0.03-0.06)	0.26 (0.17-0.41)	0.37 (−0.02-0.75)
Italy	1922	36.6 (18.6)	1953	2.14 (1.71-2.69)	0.08 (0.05-0.14)	0.47 (0.32-0.68)	0.65 (0.49-0.86)	0.44 (0.34-0.57)	0.04 (0.03-0.05)	0.30 (0.16-0.52)	0.24 (−0.08-0.63)
Spain	3499	40.8 (16.6)	3491	1.46 (1.09-1.93)	0.05 (0.03-0.08)	0.27 (0.18-0.40)	0.36 (0.22-0.53)	0.26 (0.19-0.34)	0.08 (0.05-0.10)	0.33 (0.20-0.55)	0 (−0.30-0.33)
UK	1257	43.4 (20.1)	1255	1.51 (1.14-2.01)	0.11 (0.06-0.17)	0.46 (0.30-0.65)	0.47 (0.29-0.66)	0.22 (0.17-0.30)	0.03 (0.02-0.04)	0.15 (0.09-0.24)	−0.13 (−0.42-0.20)
Netherlands	1402	48.0 (20.9)	1438	1.35 (1.01-1.78)	0.06 (0.04-0.10)	0.37 (0.24-0.52)	0.36 (0.22-0.57)	0.23 (0.16-0.30)	0.03 (0.02-0.05)	0.20 (0.12-0.33)	−0.17 (−0.46-0.14)
Germany	1974	48.4 (19.2)	1990	1.56 (1.13-2.06)	0.09 (0.05-0.16)	0.46 (0.29-0.74)	0.41 (0.26-0.58)	0.24 (0.18-0.32)	0.04 (0.03-0.06)	0.17 (0.10-0.28)	−0.03 (−0.35-0.32)
Sweden	NA	NA	929	1.41 (1.06-1.86)	0.09 (0.05-0.16)	0.42 (0.28-0.62)	0.39 (0.25-0.56)	0.23 (0.17-0.30)	0.03 (0.02-0.04)	0.13 (0.07-0.22)	NA
Denmark	2023	39.2 (20.2)	2026	1.12 (0.77-1.55)	0.08 (0.04-0.14)	0.30 (0.18-0.50)	0.32 (0.20-0.48)	0.20 (0.14-0.27)	0.02 (0.01-0.03)	0.08 (0.04-0.15)	−0.39 (−0.68-−0.08)
Age:										
<40	1219	43.6 (18.7)	1486	1.56 (1.18-2.05)	0.07 (0.04-0.12)	0.36 (0.23-0.56)	0.50 (0.34-0.70)	0.24 (0.18-0.32)	0.04 (0.03-0.07)	0.20 (0.12-0.34)	−0.01 (−0.31-0.33)
40-<60	8913	42.1 (18.7)	9509	1.54 (1.11-2.09)	0.07 (0.04-0.13)	0.37 (0.23-0.58)	0.42 (0.26-0.62)	0.26 (0.19-0.36)	0.04 (0.02-0.07)	0.21 (0.11-0.38)	−0.03 (−0.36-0.34)
≥60	2457	42.5 (21.2)	2623	1.41 (0.97-1.95)	0.08 (0.04-0.14)	0.40 (0.24-0.60)	0.32 (0.19-0.51)	0.25 (0.18-0.35)	0.03 (0.02-0.05)	0.17 (0.09-0.33)	−0.15 (−0.49-0.26)
Body mass index:										
<25	5304	45.6 (19.9)	5891	1.68 (1.22-2.26)	0.09 (0.05-0.17)	0.45 (0.29-0.71)	0.44 (0.28-0.64)	0.27 (0.20-0.38)	0.04 (0.02-0.06)	0.20 (0.11-0.36)	0.05 (−0.28-0.45)
25-<30	5070	40.6 (18.5)	5412	1.46 (1.05-1.96)	0.07 (0.04-0.12)	0.34 (0.21-0.52)	0.40 (0.24-0.59)	0.25 (0.18-0.35)	0.04 (0.02-0.07)	0.20 (0.11-0.37)	−0.09 (−0.41-0.26)
≥30	2117	38.5 (18.0)	2209	1.31 (0.92-1.81)	0.05 (0.03-0.08)	0.27 (0.18-0.43)	0.36 (0.21-0.55)	0.23 (0.17-0.31)	0.04 (0.03-0.07)	0.20 (0.10-0.38)	−0.19 (−0.50-0.16)
Sex:										
Male	4613	36.7 (17.6)	5088	1.30 (0.93-1.77)	0.06 (0.03-0.10)	0.28 (0.18-0.44)	0.40 (0.24-0.61)	0.24 (0.17-0.33)	0.04 (0.02-0.07)	0.14 (0.07-0.27)	−0.21 (−0.53-0.13)
Female	7976	45.6 (19.4)	8530	1.67 (1.22-2.24)	0.08 (0.05-0.15)	0.44 (0.28-0.67)	0.42 (0.26-0.61)	0.27 (0.19-0.37)	0.04 (0.02-0.07)	0.24 (0.13-0.42)	0.04 (−0.28-0.43)
Education†:										
Low	5174	40.3 (18.7)	5470	1.48 (1.05-2.02)	0.06 (0.03-0.10)	0.33 (0.20-0.51)	0.38 (0.22-0.59)	0.26 (0.18-0.37)	0.05 (0.03-0.08)	0.24 (0.12-0.43)	−0.05 (−0.39-0.33)
Middle	4617	42.9 (19.1)	5139	1.49 (1.08-2.05)	0.08 (0.04-0.14)	0.39 (0.24-0.61)	0.42 (0.26-0.62)	0.25 (0.18-0.35)	0.03 (0.02-0.06)	0.18 (0.10-0.31)	−0.08 (−0.40-0.29)
High	2517	45.4 (19.9)	2724	1.63 (1.19-2.16)	0.09 (0.05-0.17)	0.44 (0.27-0.69)	0.44 (0.29-0.62)	0.26 (0.19-0.35)	0.04 (0.02-0.06)	0.19 (0.11-0.34)	0 (−0.31-0.38)
Smoking status:										
Never	5866	45.1 (18.3)	6431	1.66 (1.23-2.24)	0.08 (0.05-0.15)	0.43 (0.28-0.65)	0.42 (0.26-0.61)	0.27 (0.20-0.37)	0.04 (0.03-0.07)	0.25 (0.14-0.44)	0.06 (−0.26-0.43)
Former	3359	42.6 (18.9)	3566	1.51 (1.08-2.04)	0.08 (0.04-0.14)	0.37 (0.23-0.57)	0.42 (0.27-0.62)	0.26 (0.19-0.36)	0.04 (0.02-0.06)	0.19 (0.10-0.32)	−0.07 (−0.39-0.30)
Current	3212	37.0 (20.1)	3448	1.27 (0.92-1.78)	0.05 (0.03-0.09)	0.28 (0.18-0.45)	0.39 (0.23-0.59)	0.23 (0.16-0.32)	0.04 (0.02-0.06)	0.15 (0.07-0.28)	−0.23 (−0.55-0.12)
Physical activity:										
Inactive	2953	39.1 (19.4)	3229	1.51 (1.10-2.07)	0.06 (0.04-0.11)	0.35 (0.22-0.54)	0.41 (0.24-0.61)	0.26 (0.19-0.36)	0.04 (0.03-0.07)	0.23 (0.12-0.42)	−0.06 (−0.37-0.31)
Moderately inactive	4161	42.9 (18.8)	4418	1.54 (1.10-2.08)	0.07 (0.04-0.13)	0.37 (0.23-0.59)	0.42 (0.26-0.61)	0.26 (0.19-0.35)	0.04 (0.02-0.07)	0.21 (0.11-0.38)	−0.04 (−0.37-0.33)
Moderately active	2732	43.3 (19.0)	3020	1.53 (1.10-2.08)	0.08 (0.05-0.15)	0.39 (0.24-0.62)	0.41 (0.25-0.61)	0.26 (0.18-0.36)	0.04 (0.02-0.06)	0.19 (0.10-0.34)	−0.04 (−0.37-0.36)
Active	2542	44.3 (19.5)	2745	1.50 (1.06-2.04)	0.08 (0.04-0.14)	0.38 (0.23-0.59)	0.41 (0.25-0.60)	0.25 (0.18-0.35)	0.03 (0.02-0.06)	0.18 (0.09-0.32)	−0.07 (−0.41-0.30)
Alcohol intake (g/day):										
0	2129	40.9 (18.6)	2213	1.60 (1.14-2.14)	0.06 (0.04-0.11)	0.36 (0.24-0.55)	0.39 (0.24-0.59)	0.26 (0.19-0.37)	0.05 (0.03-0.08)	0.28 (0.15-0.49)	−0.01 (−0.32-0.36)
0-<6	3885	44.9 (19.2)	4639	1.60 (1.16-2.18)	0.09 (0.05-0.16)	0.43 (0.28-0.65)	0.42 (0.25-0.62)	0.25 (0.18-0.35)	0.04 (0.02-0.06)	0.21 (0.11-0.38)	0.01 (−0.32-0.41)
6-<12	1847	43.8 (19.3)	1969	1.56 (1.12-2.10)	0.08 (0.05-0.15)	0.41 (0.26-0.63)	0.42 (0.27-0.62)	0.25 (0.18-0.35)	0.04 (0.02-0.06)	0.19 (0.10-0.33)	−0.04 (−0.38-0.33)
12-<24	2054	42.7 (19.0)	2104	1.52 (1.11-2.04)	0.07 (0.04-0.14)	0.37 (0.23-0.57)	0.43 (0.27-0.62)	0.26 (0.19-0.36)	0.04 (0.02-0.06)	0.19 (0.10-0.35)	−0.06 (−0.38-0.31)
≥24	2624	38.4 (19.2)	2643	1.31 (0.93-1.80)	0.06 (0.03-0.10)	0.27 (0.15-0.43)	0.39 (0.24-0.58)	0.25 (0.18-0.36)	0.04 (0.02-0.07)	0.16 (0.07-0.29)	−0.17 (−0.51-0.18)
Marital status:										
Single	614	46.7 (20.4)	730	1.56 (1.17-2.27)	0.09 (0.05-0.17)	0.43 (0.26-0.71)	0.47 (0.28-0.66)	0.26 (0.18-0.37)	0.04 (0.02-0.05)	0.18 (0.11-0.32)	0.02 (−0.30-0.47)
Married	5491	43.5 (19.7)	6299	1.68 (1.22-2.26)	0.09 (0.05-0.15)	0.45 (0.30-0.68)	0.47 (0.29-0.67)	0.28 (0.20-0.40)	0.04 (0.02-0.05)	0.19 (0.11-0.34)	0.02 (−0.31-0.40)
Separated/ divorced	508	47.8 (21.4)	573	1.63 (1.18-2.12)	0.09 (0.05-0.16)	0.45 (0.29-0.67)	0.44 (0.28-0.63)	0.24 (0.18-0.34)	0.04 (0.02-0.06)	0.19 (0.10-0.31)	0.02 (−0.30-0.36)
Widowed	284	42.9 (21.1)	318	1.62 (1.15-2.08)	0.09 (0.05-0.14)	0.46 (0.28-0.65)	0.41 (0.25-0.58)	0.26 (0.19-0.37)	0.03 (0.02-0.05)	0.21 (0.12-0.36)	−0.02 (−0.37-0.28)
Employment:										
No	3054	42.3 (21.0)	3313	1.55 (1.07-2.17)	0.08 (0.05-0.14)	0.43 (0.27-0.66)	0.40 (0.24-0.61)	0.27 (0.18-0.39)	0.03 (0.02-0.05)	0.19 (0.10-0.33)	−0.06 (−0.42-0.34)
Yes	5879	43.2 (19.7)	6657	1.54 (1.11-2.10)	0.09 (0.05-0.16)	0.42 (0.26-0.64)	0.44 (0.28-0.64)	0.25 (0.18-0.35)	0.03 (0.02-0.05)	0.16 (0.09-0.29)	−0.08 (−0.41-0.31)
HDL-C:‡										
Low (<1.4 mmol/L)	4862	38.5 (18.1)	5395	1.34 (0.96-1.84)	0.06 (0.04-0.11)	0.31 (0.19-0.49)	0.39 (0.24-0.59)	0.23 (0.16-0.32)	0.04 (0.02-0.06)	0.17 (0.09-0.31)	−0.19 (−0.50-0.15)
High (≥1.4 mmol/L)	7468	44.9 (19.5)	7959	1.65 (1.20-2.22)	0.08 (0.05-0.15)	0.42 (0.27-0.64)	0.43 (0.27-0.62)	0.28 (0.20-0.38)	0.04 (0.02-0.07)	0.23 (0.12-0.41)	0.04 (−0.29-0.43)
LDL-C:‡										
Low (<3.7 mmol/L)	6016	43.4 (19.0)	6545	1.44 (1.04-1.94)	0.07 (0.04-0.13)	0.35 (0.22-0.55)	0.39 (0.24-0.57)	0.24 (0.17-0.33)	0.04 (0.02-0.06)	0.19 (0.10-0.34)	−0.11 (−0.41-0.24)
High (≥3.7 mmol/L)	6169	41.6 (19.4)	6662	1.62 (1.16-2.20)	0.07 (0.04-0.13)	0.40 (0.25-0.61)	0.44 (0.27-0.65)	0.27 (0.20-0.38)	0.04 (0.02-0.07)	0.22 (0.11-0.40)	0.01 (−0.33-0.40)

HDL-C=high density lipoprotein cholesterol; IQR=interquartile range; LDL-C=low density lipoprotein cholesterol; NA=not available; SD=standard deviation.

Vitamin C was not measured in the Swedish cohort owing to sample availability.

* Distribution of the biomarkers by population characteristics was from the subcohort of EPIC-InterAct Study, except for the first two rows describing distribution among future cases and non-cases.

† Education: low=none or primary school completed; middle=technical/professional or secondary school; high=longer education (including university degree).

‡ Cut-off values for low or high levels of high density lipoprotein cholesterol and low density lipoprotein cholesterol are based on the population median in the subcohort.

### Association of plasma vitamin C, total and individual carotenoids with potential correlates

After adjustment for demographic, lifestyle, and dietary factors, positive associations were found for sex (women compared with men), physical activity, and education level with both plasma vitamin C and total carotenoids, while waist circumference and current smoking status (compared with never smoking) were inversely associated (supplemental figure S2, supplemental table S2).

**Fig 1:**
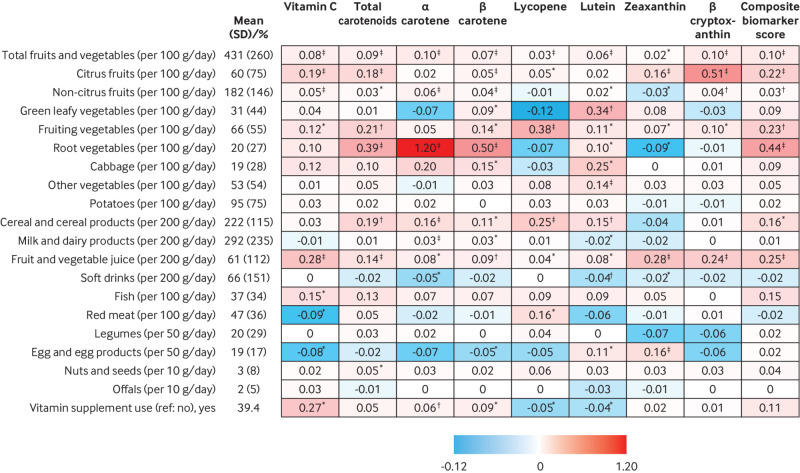
Association of dietary factors with plasma vitamin C and carotenoids in the subcohort of the European Prospective Investigation into Cancer and Nutrition (EPIC)-InterAct study. The values in the box represent the differences in vitamin C, carotenoids, or the composite biomarker score (in standard deviaiton units) for each one standardised unit per category difference in dietary factors. The mean (standard deviation) or percentage of participants for the examined dietary factors are presented in the second column. Linear regression was used to obtain the country specific estimate of an association, adjusting for demographic, lifestyle, and other examined dietary factors if available in that country, except the overlapping food groups. The country-specific estimates were then combined using random effects meta-analysis. *P<0.05; †P<0.001; ‡P<0.0001. Sample size was 10 584 for vitamin C and 11 537 for the carotenoid estimation. All the values are expressed in a red scale for different levels of positive associations and blue scale for different levels of negative associations. All the carotenoids variables were natural log transformed before statistical analysis in the linear regression. Standard deviation was 19.2 µmol/L for vitamin C, 0.50 for total carotenoids (log transformed), 0.91 for α carotene (log transformed), 0.73 for β carotene (log transformed), 0.68 for lycopene (log transformed), 0.51 for lutein (log transformed), 0.91 for zeaxanthin (log transformed), 0.94 for β cryptoxanthin (log transformed), and 0.57 for the composite biomarker score. A β coefficient of 0.10 represents the increase in the composite biomarker score per 100 g/day of fruit and vegetable intake. Performing this cross sectional analysis the other way round, every change in one standard deviation in the composite biomarker score was associated with a 66 g/day increase in fruit and vegetable intake

Dietary intake of total fruits and vegetables, citrus fruits, non-citrus fruits, fruiting vegetables, and fruit and vegetable juice were all positively associated with both plasma vitamin C, total carotenoids, and the composite biomarker score (fig 1). Each 100 g/day higher intake of fruits and vegetables was associated with 0.10 higher level of the composite biomarker score (fig 1); conversely, every one standard deviation higher composite biomarker score was associated with a 66 (95% confidence interval 61 to 71) g/day higher intake of fruits and vegetables. The median fruit and vegetable intake was 274, 357, 396, 452, and 508 g/day for levels 1 (lowest) to 5 (highest) of the composite biomarker score, respectively (table 2). Forest plots showing the association of fruit and vegetable intake subgroups with each plasma biomarker by country and overall are presented in supplemental figure S3; the directions of these associations were generally consistent across countries.

**Table 2:** Prospective associations between plasma vitamin C and carotenoids and incident type 2 diabetes in the EPIC-InterAct study*

	Hazard ratio (95% confidence interval)†						P value for trend
	Group 1	Group 2	Group 3	Group 4	Group 5	For each one standard deviation	
Vitamin C, (median, µmol/L)	17.0	32.8	42.3	51.2	64.9	—	—
No of cases/person years	2431/13 089	2042/13 166	1482/13 337	1256/14 898	949/17 420	—	—
Model 1a	1.0 (ref)	0.84 (0.77 to 0.92)	0.61 (0.53 to 0.71)	0.53 (0.46 to 0.61)	0.39 (0.31 to 0.50)	0.71 (0.65 to 0.78)	<0.001
Model 1b	1.0 (ref)	0.87 (0.80 to 0.96)	0.65 (0.56 to 0.76)	0.57 (0.50 to 0.66)	0.43 (0.34 to 0.54)	0.74 (0.68 to 0.80)	<0.001
Model 2	1.0 (ref)	0.94 (0.83 to 1.08)	0.72 (0.60 to 0.87)	0.68 (0.56 to 0.82)	0.58 (0.47 to 0.72)	0.82 (0.76 to 0.89)	<0.001
Total carotenoids, (median, µmol/L)	0.75	1.17	1.52	1.93	2.70	—	—
No of cases/person years	2952/10 116	1944/11 940	1316/14 155	1127/16 904	874/19 899	—	—
Model 1a	1.0 (ref)	0.67 (0.61 to 0.74)	0.43 (0.37 to 0.52)	0.35 (0.28 to 0.42)	0.24 (0.18 to 0.32)	0.53 (0.47 to 0.61)	<0.001
Model 1b	1.0 (ref)	0.72 (0.62 to 0.83)	0.51 (0.44 to 0.60)	0.43 (0.36 to 0.52)	0.31 (0.24 to 0.40)	0.60 (0.53 to 0.67)	<0.001
Model 2	1.0 (ref)	0.83 (0.74 to 0.92)	0.64 (0.54 to 0.75)	0.61 (0.53 to 0.70)	0.51 (0.43 to 0.60)	0.75 (0.68 to 0.82)	<0.001
α carotene, (median, µmol/L)	0.02	0.05	0.07	0.12	0.23	—	—
No of cases/person years	2629/10 061	1922/13 665	1571/15 458	1254/16 266	837/17 564	—	—
Model 1a	1.0 (ref)	0.72 (0.65 to 0.80)	0.54 (0.46 to 0.62)	0.46 (0.41 to 0.52)	0.29 (0.24 to 0.35)	0.57 (0.48 to 0.68)	<0.001
Model 1b	1.0 (ref)	0.75 (0.66 to 0.86)	0.60 (0.52 to 0.69)	0.53 (0.47 to 0.60)	0.36 (0.30 to 0.43)	0.65 (0.56 to 0.76)	<0.001
Model 2	1.0 (ref)	0.81 (0.70 to 0.94)	0.72 (0.64 to 0.82)	0.69 (0.59 to 0.79)	0.53 (0.46 to 0.62)	0.79 (0.72 to 0.88)	<0.001
β carotene, (median, µmol/L)	0.14	0.26	0.37	0.53	0.87	—	—
No of cases/person years	2911/8142	2064/11 861	1417/15 096	1035/17 520	786/20 395	—	—
Model 1a	1.0 (ref)	0.68 (0.60 to 0.78)	0.44 (0.40 to 0.49)	0.33 (0.28 to 0.37)	0.24 (0.20 to 0.28)	0.48 (0.40 to 0.58)	<0.001
Model 1b	1.0 (ref)	0.70 (0.61 to 0.80)	0.48 (0.43 to 0.54)	0.38 (0.34 to 0.43)	0.29 (0.26 to 0.34)	0.55 (0.46 to 0.65)	<0.001
Model 2	1.0 (ref)	0.78 (0.69 to 0.88)	0.60 (0.54 to 0.68)	0.53 (0.47 to 0.60)	0.45 (0.39 to 0.52)	0.71 (0.62 to 0.82)	<0.001
Lycopene, (median, µmol/L)	0.15	0.28	0.41	0.56	0.82	—	—
No of cases/person years	2395/10 449	1879/12 779	1447/13 840	1299/16 393	1193/19 553	—	—
Model 1a	1.0 (ref)	0.79 (0.69 to 0.90)	0.66 (0.60 to 0.73)	0.57 (0.48 to 0.69)	0.50 (0.38 to 0.64)	0.76 (0.68 to 0.85)	<0.001
Model 1b	1.0 (ref)	0.89 (0.80 to 0.99)	0.73 (0.65 to 0.82)	0.68 (0.59 to 0.78)	0.59 (0.47 to 0.75)	0.81 (0.75 to 0.89)	<0.001
Model 2	1.0 (ref)	0.95 (0.86 to 1.06)	0.84 (0.75 to 0.94)	0.81 (0.71 to 0.92)	0.79 (0.68 to 0.93)	0.91 (0.85 to 0.98)	0.01
Lutein, (median, µmol/L)	0.13	0.20	0.25	0.33	0.49	—	—
No of cases/person years	2508/11 727	1856/11 950	1464/13 668	1268/14 951	1117/2 0718	—	—
Model 1a	1.0 (ref)	0.72 (0.66 to 0.79)	0.52 (0.44 to 0.61)	0.41 (0.32 to 0.51)	0.30 (0.24 to 0.38)	0.57 (0.48 to 0.68)	<0.001
Model 1b	1.0 (ref)	0.79 (0.72 to 0.87)	0.64 (0.57 to 0.71)	0.56 (0.48 to 0.65)	0.49 (0.42 to 0.56)	0.70 (0.62 to 0.79)	<0.001
Model 2	1.0 (ref)	0.89 (0.78 to 1.01)	0.75 (0.65 to 0.86)	0.70 (0.59 to 0.82)	0.65 (0.55 to 0.78)	0.84 (0.77 to 0.91)	<0.001
Zeaxanthin, (median, µmol/L)	0.01	0.03	0.04	0.06	0.10	—	—
No of cases/person years	2159/13 624	1658/17 107	1510/18 591	1479/15 470	1407/8222	—	—
Model 1a	1.0 (ref)	0.87 (0.74 to 1.01)	0.77 (0.66 to 0.90)	0.70 (0.58 to 0.85)	0.55 (0.47 to 0.65)	0.76 (0.68 to 0.85)	<0.001
Model 1b	1.0 (ref)	0.92 (0.78 to 1.09)	0.89 (0.78 to 1.01)	0.84 (0.67 to 1.05)	0.71 (0.59 to 0.86)	0.87 (0.80 to 0.94)	<0.001
Model 2	1.0 (ref)	0.95 (0.84 to 1.07)	0.98 (0.86 to 1.13)	0.99 (0.78 to 1.27)	0.86 (0.66 to 1.12)	0.96 (0.88 to 1.05)	0.34
β cryptoxanthin, (median, µmol/L)	0.05	0.12	0.20	0.32	0.61	—	—
No of cases/person years	2431/10 990	1712/15 889	1494/16 483	1379/15 438	1197/14 213	—	—
Model 1a	1.0 (ref)	0.75 (0.63 to 0.89)	0.63 (0.52 to 0.77)	0.58 (0.49 to 0.69)	0.42 (0.31 to 0.56)	0.64 (0.55 to 0.75)	<0.001
Model 1b	1.0 (ref)	0.77 (0.65 to 0.91)	0.74 (0.64 to 0.87)	0.66 (0.56 to 0.78)	0.51 (0.38 to 0.69)	0.75 (0.67 to 0.84)	<0.001
Model 2	1.0 (ref)	0.81 (0.68 to 0.97)	0.81 (0.69 to 0.95)	0.83 (0.72 to 0.96)	0.72 (0.56 to 0.93)	0.88 (0.81 to 0.96)	0.001
Composite biomarker score (median)	−0.66	−0.31	−0.05	0.23	0.74	—	—
Fruit and vegetable intake (median, g/day)	274	357	396	452	508	—	—
No of cases/person years	2752/10 909	1719/13 249	1249/14 624	1047/15 582	770/17 471	—	—
Model 1a	1.0 (ref)	0.61 (0.53 to 0.70)	0.43 (0.35 to 0.53)	0.32 (0.24 to 0.43)	0.22 (0.17 to 0.30)	0.55 (0.48 to 0.63)	<0.001
Model 1b	1.0 (ref)	0.67 (0.59 to 0.77)	0.52 (0.44 to 0.62)	0.42 (0.33 to 0.52)	0.31 (0.25 to 0.39)	0.61 (0.55 to 0.69)	<0.001
Model 2	1.0 (ref)	0.77 (0.68 to 0.87)	0.66 (0.54 to 0.80)	0.59 (0.48 to 0.72)	0.50 (0.40 to 0.62)	0.75 (0.67 to 0.83)	<0.001

* Sample size is 19 255 for vitamin C, 19 907 for carotenoids, and 18 276 for the composite biomarker score. Numbers (N) of cases and person years (subcohort) by the biomarker split into five equal groups (group 1, the lowest; group 5, the highest) are presented. Model 1a: adjusted for age (as underlying timescale), sex and centre; model 1b: model 1a+physical activity, smoking status, employment, marital status, education, alcohol intake, total energy intake, high density lipoprotein cholesterol, and low density lipoprotein cholesterol; model 2: model 1b+adiposity (body mass index and waist circumference). Model 1b and model 2 of plasma vitamin C were not adjusted for high and low density lipoprotein cholesterol.

† Hazard ratios for groups 1-4 (compared with group 1) and for each one standard deviation (SD) of each biomarker, were estimated from country-specific, Prentice weighted Cox regression models; estimates were then combined across countries by random effects meta-analysis. P value for trend was calculated as the trend per group for each biomarker. The plasma composite biomarker score was generated by calculating the average of the z scores of vitamin C and six individual carotenoids. Standard deviation was 19.2 µmol/L for vitamin C, 0.83 µmol/L for total carotenoids, 0.12 µmol/L for α carotene, 0.41 µmol/L for β carotene, 0.27 µmol/L for lycopene, 0.15 µmol/L for lutein, 0.04 µmol/L for zeaxanthin, 0.29 µmol/L for β cryptoxanthin, and 0.57 for the composite biomarker score.

### Association of plasma vitamin C, total and individual carotenoids with incident type 2 diabetes

Higher levels of plasma vitamin C were associated with a lower hazard of type 2 diabetes; hazard ratio for each standard deviation 0.71 (95% confidence interval 0.65 to 0.78) in a model adjusted for age, sex, and study centre (model 1a). Hazard ratios comparing groups 2, 3, 4, and 5 of vitamin C with group 1 (the lowest group) were 0.84 (0.77 to 0.92), 0.61 (0.53 to 0.71), 0.53 (0.46 to 0.61), and 0.39 (0.31 to 0.50). These inverse associations were attenuated after further adjustment for socioeconomic factors, lifestyle factors, and adiposity (model 2), but the trend across the groups remained: hazard ratios 0.94 (0.83 to 1.08), 0.72 (0.60 to 0.87), 0.68 (0.56 to 0.82), and 0.58 (0.47 to 0.72) comparing groups 2, 3, 4, and 5, with group 1, respectively (table 2).

For plasma total carotenoids, the inverse association with type 2 diabetes estimated in models 1a and 1b was attenuated after further adjustment for adiposity (model 2), but remained significant. The hazard ratio for each standard deviation of total carotenoids was 0.75 (95% confidence interval 0.68 to 0.82). The hazard ratios comparing groups 2, 3, 4, and 5 of total carotenoids with group 1 were 0.83 (0.74 to 0.92), 0.64 (0.54 to 0.75), 0.61 (0.53 to 0.70), and 0.51 (0.43 to 0.60), respectively. In model 2, all plasma individual carotenoids were inversely associated with type 2 diabetes, except for zeaxanthin (table 2). For plasma total and individual carotenoids, the heterogeneity (I^2^) across countries ranged from 48.0% for β cryptoxanthin to 75.2% for β carotene (supplemental figure S4), but the directions of the associations were consistent across different countries.

The composite biomarker score, which included contributions from all the examined biomarkers, was inversely associated with type 2 diabetes. The hazard ratios comparing groups 2, 3, 4, and 5 of the combined biomarker score with group 1 were 0.77 (0.68 to 0.87), 0.66 (0.54 to 0.80), 0.59 (0.48 to 0.72), and 0.50 (0.40 to 0.62), respectively. One standard deviation difference in the composite biomarker score, equivalent to a 66 (95% confidence interval 61 to 71) g/day difference in total fruit and vegetable intake, was associated with a hazard ratio of 0.75 (95% confidence interval 0.67 to 0.83). This would be equivalent to an absolute risk reduction of 0.95 per 1000 person years of follow-up if achieved across an entire population with the characteristics of the eight European countries included in this analysis. Thus the crude overall incidence rate of type 2 diabetes would be estimated to be reduced from 3.8 to 2.85 per 1000 person years of follow-up in this population. 

**Fig 2:**
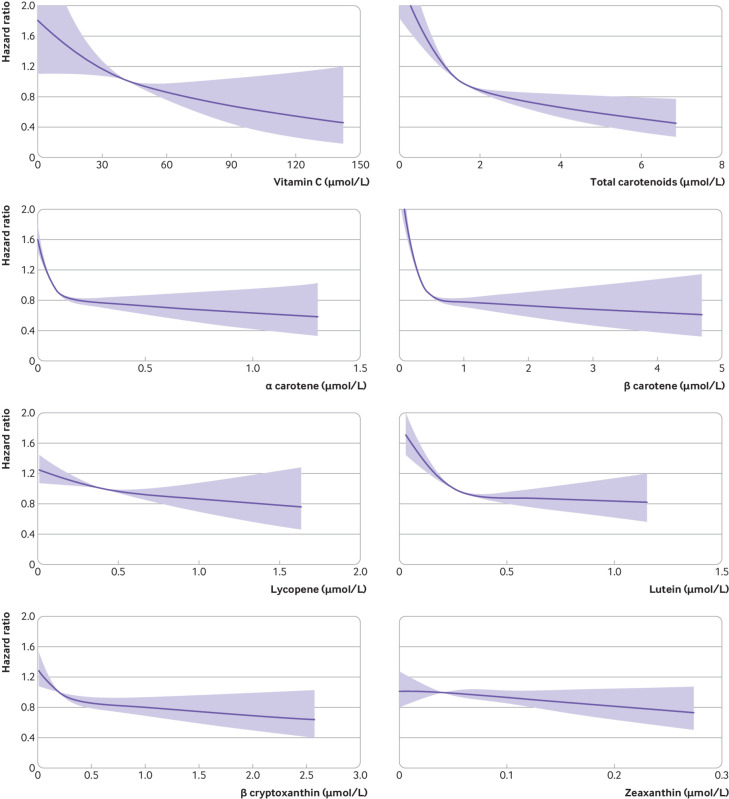
Associations of plasma vitamin C and carotenoids with incident type 2 diabetes in the European Prospective Investigation into Cancer and Nutrition (EPIC)-InterAct Study. The purple solid line and the shaded area represent estimates of hazard ratios and the 95% confidence intervals, respectively, for each biomarker (median in the subcohort as a reference). Covariates included age (as underlying time scale), sex, centre, physical activity, smoking status, employment, marital status, education, alcohol intake, total energy intake, high density lipoprotein cholesterol (for analyses of carotenoids only), low density lipoprotein cholesterol (for analyses of carotenoids only), body mass index, and waist circumference. For total carotenoids, α carotene, β carotene, and lutein, we found evidence of a non-linear association (P value for non-linearity <0.001)

**Fig 3:**
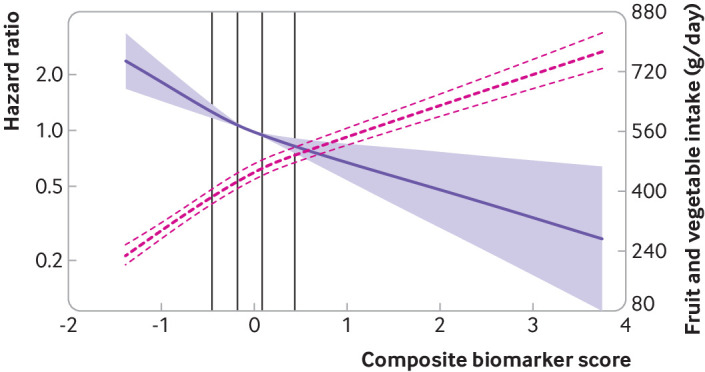
Associations of plasma composite biomarker score with incident type 2 diabetes and total fruit and vegetable intake in the European Prospective Investigation into Cancer and Nutrition (EPIC)-InterAct Study. The purple solid line and shaded areas represent hazard ratios and 95% confidence intervals for the association of the composite biomarker score with type 2 diabetes (median as a reference), adjusted for age (as underlying time scale), sex, centre, physical activity, smoking status, employment, marital status, education, alcohol intake, total energy intake, high density lipoprotein cholesterol, low density lipoprotein cholesterol, body mass index, and waist circumference. The red dashed lines represent the association of the composite biomarker score with fruit and vegetable intake (best estimates and their 95% confidence intervals), adjusted for age, sex, centre, physical activity, smoking status, employment, marital status, education, alcohol intake, total energy intake, body mass index, waist circumference, and dietary intake of potatoes, cereal and cereal products, milk and dairy products, fruit and vegetable juice, soft drinks, fish, red meat, legumes, egg and egg products, nuts and seeds, offals, and vitamin supplement use. P values for non-linearity were 0.056 for the hazard ratios, and <0.001 for estimates for fruit and vegetable intake. The vertical lines within the figure represent the quintiles (20th, 40th, 60th, and 80th centiles) of the composite biomarker score in the subcohort

We found evidence of non-linear associations (P value for non-linearity <0.001) for plasma total carotenoids, α carotene, β carotene, and lutein, with a strong inverse association with type 2 diabetes at low to moderate concentrations, but weaker at higher concentrations (fig 2). The shape of the associations of the composite biomarker score with total fruit and vegetable intake and with type 2 diabetes is presented in figure 3. In an analysis of the association with type 2 diabetes using this biomarker score as a cut-off level to define consumption of five or more portions a day of fruits and vegetables, the hazard ratio compared with consuming an estimated fewer than five portions a day was 0.69 (95% confidence interval 0.63 to 0.76).

In a sensitivity analysis, after mutual adjustment for the other individual biomarkers, the inverse association with type 2 diabetes for each of the biomarkers was attenuated (supplemental table S3). Lycopene and β cryptoxanthin were no longer significantly associated with type 2 diabetes. Other sensitivity analyses had little effect on the findings (supplemental tables S3 and S4).

We identified evidence of a significant interaction only between baseline age and both plasma vitamin C and α carotene (P value for interaction <0.001), with the inverse associations with type 2 diabetes being weaker in the oldest age group (supplemental figure S5).

## Discussion

In this large study with dietary diversity across eight European countries, higher concentrations of plasma vitamin C and carotenoids were associated with a lower incidence of type 2 diabetes. A composite biomarker score, comprising the seven examined biomarkers, was associated with consumption of fruit and vegetables and was also inversely related to the risk of type 2 diabetes. These findings provide strong evidence from objectively measured biomarkers for the recommendation that fruit and vegetable intake should be increased to prevent type 2 diabetes.

### Strengths and limitations of this study

The strengths of our study include our report of the association of plasma vitamin C and individual carotenoids with type 2 diabetes in a large case-cohort study. The study included a subcohort of 13 662 individuals and 9754 participants with incident type 2 diabetes, from an original cohort of 340 234 participants with 3.99 million person years of follow-up. This analysis, based on predominantly white participants from eight European countries, indicates the generalisability of the study findings to populations of European descent, though further research in other populations is warranted. The number of participants with incident type 2 diabetes in this study was 10-fold greater than in previous studies of the association of biomarkers of fruit and vegetable intake with risk of type 2 diabetes, which not only provided high statistical power for the main effect analyses but also permitted a dose-response analysis and other sensitivity analyses.

This study has several limitations. The biomarkers could degrade during storage over years, which would make it difficult to quantify the absolute concentration of vitamin C and carotenoids. The likelihood of degradation in this study was minimised, however, by storing the samples in liquid nitrogen and if it did occur, it should be the same in cases and non-cases. The vitamin C and carotenoid biomarkers were measured only at baseline, and intra-individual variation over time is likely. However, when follow-up was restricted to the first 8 years, the associations remained similar. Another limitation is the observational nature of this study and the potential for residual confounding due to mis-measured or unmeasured confounders. The inverse associations we observed could, in part, be explained by the confounding effect of a healthy lifestyle and favourable socioeconomic conditions, which might not have been fully accounted for in the analyses. Moreover, differential misclassification in ascertainment of type 2 diabetes could have occurred if those with high fruit and vegetable consumption were more health conscious, sought medical assessment, and hence were diagnosed with the disorder. This possibility could cause bias towards the null and an underestimation of the inverse association. The results could be explained by reverse causality, but our sensitivity analysis indicated that this was unlikely. An assessment of causal associations of these nutritional biomarkers with type 2 diabetes was beyond the scope of our study. In addition, a common limitation for nutritional biomarkers is that their relationship with diet is often influenced by both genetic variation and nutrient metabolism.^32^ Further challenges in interpretation arise. Thus the presence of these biomarkers might not be exclusively due to the consumption of fruit and vegetables, but some carotenoids, for instance, could also originate from the food production process, such as food colouring. The biomarkers could reflect underlying dietary patterns or food substitution effects when fruit or vegetables replace meat in the diet, for instance.

### Comparison with other studies

Evidence from previous studies for the magnitude of the association between circulating vitamin C and carotenoids and the risk of type 2 diabetes is limited, mainly because of the small number of participants in previous studies.^13^ ^14^ ^16^ ^17^ Additionally, those studies did not investigate differences in those associations by country. Biomarkers can be used not only to investigate the relationship between total fruit and vegetables and the risk of diabetes but can also help to examine the effect of specific fruit and vegetable subgroups. For example, α carotene and β carotene were highly correlated with root vegetables, in this and a previous study.^8^ Therefore, the biomarker finding of α carotene and β carotene could suggest a potential inverse association with type 2 diabetes for root vegetables; this deserves further study. In this study, the inverse association between some biomarkers and type 2 diabetes was attenuated when mutually adjusted for the other individual biomarkers. These results suggest that a combination of nutrients in fruit and vegetables explains the association with type 2 diabetes rather than a single nutrient. Thus our finding of an inverse association between the biomarker composite score and risk of type 2 diabetes could be capturing this combined effect.

Our results could also provide a possible explanation of the previous null findings of randomised trials for single vitamin supplements. In the Women’s Antioxidant Cardiovascular Study, dietary supplements of vitamin C, vitamin E, or β carotene had no significant effects on the risk of developing type 2 diabetes during a follow-up of 9.2 years.^33^ Similarly, long term β carotene supplementation did not affect the risk of incident type 2 diabetes among healthy men in a randomised trial over a 12 year follow-up period,^34^ or in male smokers in a randomised trial with 6.1 years of follow-up.^35^ The absence of a significant intervention effect on the risk of developing type 2 diabetes in these trials could have been because they focused on supplementation of these individual nutrients themselves rather than the foods or a dietary pattern for which these nutrients are objective biomarkers. Fruits and vegetables contain many other components besides vitamin C and carotenoids, including fibre, phytochemicals such as flavonoids, and other antioxidants, all of which could have beneficial health effects.^36^ Alternatively, the null results for vitamin supplementation trials could be explained by the focus of those trials on individual rather than composite nutrients, or their focus on increasing average levels rather than reducing deficiency. We observed non-linear associations for carotenoids, and thus the effect of supplementation on type 2 diabetes could be greater in those who had lower levels at baseline. Finally, the distribution of vitamin levels differed in those trials from that in our observational study. We found a mean concentration of plasma β carotene of 0.47 µmol/L. This result contrasts with a mean of 2.24 µmol/L in the Women’s Antioxidant Cardiovascular Study^33^ and 5.6 µmol/L in the Alpha-Tocopherol, Beta-Carotene Cancer Prevention Study,^35^ after β carotene intervention of 50 mg every other day or 20 mg each day, respectively.

The potential mechanisms of association could be that consumption of fruit and vegetables helps to regulate weight and adiposity, as well as glucose-insulin homoeostasis and the inflammatory status of the participants,^37^ ^38^ thus leading to a reduced risk of type 2 diabetes. Another possible mechanism is the influence of the fruit and vegetable intake on the gut microbiota, given their high concentration of fibre or other gut microbiota-related nutrients.^39^ ^40^ A sensitivity analysis of additional adjustment for a diet quality score did not alter the findings for any of the biomarkers, which further confirms that higher fruit and vegetable intake is likely to be beneficial for prevention of type 2 diabetes regardless of the overall diet quality. We could not rule out the possibility, however, that the observed association partly reflected metabolic effects independent of dietary factors.

### What this study adds

Various dietary guidelines have recommended increasing fruit and vegetable intake as an important component of a healthy diet. However, evidence derived from a food frequency questionnaire for the specific role of fruit and vegetables and their subtypes in the prevention of type 2 diabetes has previously been weak and inconsistent.^3^ ^4^ ^41^ The potential overall benefits of fruits and vegetables have also been questioned within certain popular dietary regimens that favour low carbohydrate intake, including advice to limit the consumption of many fruits and vegetables.^42^ ^43^ Although five portions a day of fruit and vegetables have been recommended for decades, in 2014-15, 69% of UK adults ate fewer than this number,^44^ and this proportion is even higher in European (EU) adults (86%).^45^ The low population level concordance with the “five a day” recommendation provides an incentive to quantify the benefits of making small changes in consumption of fruits and vegetables even below the threshold of the widely recommended guideline level, as suggested by our findings from the dose-response relationship of the biomarkers with fruit and vegetable intake presented in figure 3. Using biomarkers to quantify fruit and vegetable intake is an adjunct to self-reported questionnaires as objective assessment methods are less affected by the measurement errors of subjective dietary assessment tools. The use of biomarkers in this context is at present a research tool used to advance scientific understanding rather than a suggestion for their use in clinical practice.

This study also extends our understanding of the correlates of plasma vitamin C, total and individual carotenoids in different European countries. Previous studies,^8^ ^46^ ^47^ based on a subsample of about 3000 people in the EPIC cohort, reported that the strongest predictors of individual carotenoids were fruits for β cryptoxanthin, total carrots and root vegetable for α carotene, and tomato products for lycopene. Our findings with a larger subcohort of people from the EPIC study are broadly consistent with the previous work, and provide additional information about the correlates of vitamin C, another strong biomarker of fruit and vegetable intake. In addition to the association of plasma vitamin C and carotenoids with fruit and vegetable intake, we have also described their association with other food groups, and demographic and lifestyle factors. We found a positive association between consumption of fruit and vegetable juice and the composite biomarker score, which could reflect a combination of factors, including naturally occurring vitamin C and the content of carotenoids in juice as well as fortified products. We could not distinguish between these, but our analyses of association between the biomarkers and incident type 2 diabetes were adjusted for vitamin supplement use, and suggest that as biomarkers of fruit and vegetable intake these findings endorse the consumption of fruit and vegetables, not that of supplements.

### Conclusions

Higher levels of plasma vitamin C, total and individual carotenoid biomarkers, and their composite biomarker score were associated with a lower incidence of type 2 diabetes in diverse European populations in eight countries. These biomarkers are indicators of fruit and vegetable consumption, reducing the measurement error and bias of dietary self-reports. Our findings suggest that higher fruit and vegetable consumption is inversely associated with the incidence of type 2 diabetes, regardless of whether this increase in consumption is from a level below or above the recommended five-a-day threshold. The public health implication of this observation is that the consumption of even a moderately increased amount of fruit and vegetables among populations who typically consume low levels could help to prevent type 2 diabetes. It should be noted that these findings and other available evidence suggest that fruit and vegetable intake, rather than vitamin supplements, is potentially beneficial for the prevention of type 2 diabetes.

**What is already known on this topic**

Investigation of a link between fruit and vegetable intake and the risk of type 2 diabetes has relied on self-reported dietary questionnaires, with inconsistent findings
Evidence from objective markers of fruit and vegetable intake is sparse but would be complementary to self-report

**What this study adds**

Results from the assessment of individual blood biomarkers and a composite biomarker score comprising plasma vitamin C and carotenoids indicate an inverse association with new onset type 2 diabetes in a pan-European population
This study suggests that even a modest increase in fruit and vegetable intake could help to prevent type 2 diabetes, indicated by objective biomarkers of consumption, regardless of whether the increase is among people with initially low or high intake

## Web extra

Extra material supplied by authors

Web appendix: Online supplemental files

## Notes

Contributors: NGF and NJW contributed equally to this work. NJW was chief investigator and NGF and SJS were investigators of the InterAct project. J-SZ and NGF had full access to all the data in the study and take responsibility for the integrity of the data and the accuracy of the data analysis; J-SZ performed the statistical analyses and drafted the manuscript with input from NGF. The working group (J-SZ, SJS, FI, TEG, MS, RC, YTvdS, IS, AB, NGF) and all authors contributed to interpretation of data, revised the article critically for important intellectual content, and approved the final version of the manuscript. NGF and NJW are the guarantors, and they contributed equally to the work. The corresponding author attests that all listed authors meet authorship criteria and that no others meeting the criteria have been omitted.

Ethical approval: All included studies were approved by local review boards, and all participants gave written informed consent to participate in the study.

Data sharing: EPIC-InterAct Study data cannot be deposited publicly as these collaborative data originate from multiple research institutions across eight European countries with different legal frameworks. The authors confirm that researchers seeking the analysis dataset for this work can submit a data request to the EPIC-InterAct study central contact point by emailing interact@mrc-epid.cam.ac.uk.

The corresponding author affirms that the manuscript is an honest, accurate, and transparent account of the study being reported; that no important aspects of the study have been omitted; and that any discrepancies from the study as planned (and, if relevant, registered) have been explained.

Dissemination to participants and related patient and public communities: Findings will be disseminated via the communications team of the corresponding author’s institute.
