# Supplementary material for: Association of plasma biomarkers of fruit and vegetable intake with incident type 2 diabetes: EPIC-InterAct case-cohort study in eight European countries
Source: BMJ. 2020 Jul 8;370:m2194. doi: 10.1136/bmj.m2194 (PMC7341350; doi:10.1136/bmj.m2194)

Online Supplemental Files

"Association of plasma biomarkers of fruit and vegetable intake with incident type 2 diabetes: The EPIC-InterAct case-cohort study in eight European countries"

Supplemental Tables

Supplemental Table S1 Pairwise correlation of plasma vitamin C and carotenoids in the EPIC-InterAct study

Supplemental Table S2 Associations of demographic, lifestyle and dietary factors with plasma vitamin C and carotenoids in the EPIC-InterAct Study

Supplemental Table S3 Sensitivity analysis for the association between plasma vitamin C and carotenoids and type 2 diabetes in the EPIC-InterAct Study

Supplemental Table S4 Sensitivity analysis for the influence of individual biomarkers on the results of the composite biomarker score

Supplemental Figures

Supplemental Figure S1 EPIC-InterAct study design and participants included in the analysis.

Supplemental Figure S2 Summary of the association of demographic and lifestyle factors with plasma vitmain C and carotenoids in the subcohort of EPIC-InterAct study. The values in the box represent the differences in vitamin C and total carotenoids (in SD unit) per 1-standardised unit/category difference in each demographic or lifestyle factors. The mean (SD) or % of participants for the examined demographic, lifestyle factors were presented in the second column. Linear regression was used to obtain the country-specific estimate of an association with mutual adjustment for all the other examined demographic, lifestyle factors if available in that country. The country-specific estimates were then combined using random effects meta-analysis. *indicates $p<0.05$, ** indicates $p<0.001$, *** indicates $p<0.0001$. Sample size was 10,584 for vitamin C and 11,537 for the carotenoids estimation. All the values are expressed in red scale for different levels of positive associations and blue scale for different levels of negative associations. All of the carotenoids variables were natural log-transformed before statistical analysis in the linear regression. SD was 19.2 µmol/L for vitamin C, 0.50 for total carotenoids (ln-transformed), 0.91 for α-carotene (ln-transformed), 0.73 for β-carotene (ln-transformed), 0.68 for lycopene (ln-transformed), 0.51 for lutein (ln-transformed), 0.91 for zeaxanthin (ln-transformed), 0.94 for β-cryptoxanthin (ln-transformed) and 0.57 for CB-score (composite biomarker score).

Supplemental Figure S3 Association of dietary fruit and vegetable with plasma vitmain C and carotenoids stratified by country in the subcohort of EPIC-InterAct study. The x-axis values in the figure represent standardised difference (error bar: 95%CI) in vitamin C and carotenoids (in SD unit) per 100 g/d difference in each fruit and vegetable subgroup. Linear regression was used to obtain the country specific estimate of an association with mutual adjustment for all the other examined demographic, lifestyle or dietary factors if available in that country. The country-specific estimates were then combined using random effects meta-analysis. All of the carotenoids variables were natural log-transformed before statistical analysis in the linear regression. SD was 19.2 µmol/L for vitamin C, 0.50 for total carotenoids (ln-transformed), 0.91 for α-carotene (ln-transformed), 0.73 for β-carotene (ln-transformed), 0.68 for lycopene (ln-transformed), 0.51 for lutein (ln-transformed), 0.91 for zeaxanthin (ln-transformed), 0.94 for β-cryptoxanthin (ln-transformed) and 0.57 for composite biomarker score. Citrus fruits included grapefruit, orange, tangerine, lemon; Non-citrus fruits included apple, pear, grape, apricot, cherry, peach, plum, nectarine, prune, melon, pineapple, strawberry, raspberry, banana, kiwi; green leafy vegetables included spinach, chard, endive, lettuce, borage, watercress, beet leaves; fruiting vegetables included tomato, pepper, avocado, courgette, artichoke, aubergine, pumpkin, squash; root vegetables included carrot, radish, salsify, beetroot, turnip, celeriac, swede; cabbage included broccoli, cabbage, brussel sprouts, cauliflower, kale; other vegetables: vegetables not included in the above groups.

Supplemental Figure S4 Association of plasma vitamin C and carotenoids (per 1-standard deviation) with type 2 diabetes by countries in the EPIC-InterAct study. Hazard Ratios (HRs) are per 1-standard deviation (SD) of each biomarker, estimated from country-specific Prentice-weighted Cox regression models, with adjustment for age (as underlying timescale), sex, centre, physical activity, smoking status, employment, marital status, education, alcohol intake, total energy intake, HDL-C (not for vitamin C), LDL-C (not for vitamin C), BMI and waist circumference; estimates were then combined across countries using random-effects meta-analysis. SD was 19.2 µmol/L for vitamin C, 0.83 µmol/L for total carotenoids, 0.12 µmol/L for α-carotene, 0.41 µmol/L for β-carotene, 0.27µmol/L for lycopene, 0.15 µmol/L for lutein, 0.04 µmol/L for zeaxanthin, 0.29 µmol/L for β-cryptoxanthin, and 0.57 for composite biomarker score.

Supplemental Figure S5 Association between plasma vitamin C, carotenoids and type 2 diabetes (per 1-standard deviation) stratified by baseline age, BMI and vitamin supplement subgroups in the EPIC-InterAct study. Interaction of plasma vitamin C and carotenoids with baseline age, sex, BMI, physical activity, smoking status, season and vitamin supplements for type 2 diabetes was examined and the interactions with p-value<0.05 were presented. * indicated that the p-value for the interaction analysis passed the Bonferroni correction (p-interaction threshold for the multiple correction= 0.0008). Values were presented as hazard ratio (95%CI) of type 2 diabetes per 1-standard deviation (SD) difference in each biomarker stratified by the age, BMI or vitamin supplement subgroup. The statistical model in the stratified analysis was the same as the model 2 except that the samples were divided into several subgroups, adjusted for age (as underlying timescale), sex, centre, physical activity, smoking status, employment, marital status, education, alcohol intake, total energy intake, HDL-C (not for vitamin C), LDL-C (not for vitamin C), BMI and waist circumference. SD was 19.2 µmol/L for vitamin C, 0.83 µmol/L for total carotenoids, 0.12 µmol/L for α-carotene, 0.41 µmol/L for β-carotene, 0.27µmol/L for lycopene, 0.15 µmol/L for lutein, 0.04 µmol/L for zeaxanthin, 0.29 µmol/L for β-cryptoxanthin, and 0.57 for composite biomarker score.

Supplemental Table S1 Pairwise correlation of plasma vitamin C and carotenoids in the EPIC-InterAct study*

	Mean Spearman correlation coefficients (minimum, maximum) across eight countries †							
	Vitamin C	Total carotenoids	α-carotene	β-carotene	Lycopene	Lutein	Zeaxanthin	β-cryptoxanthin
Vitamin C	1							
Total carotenoids	0.36 (0.28, 0.42)	1						
α-carotene	0.28 (0.14, 0.36)	0.66 (0.47, 0.74)	1					
β-carotene	0.33 (0.25, 0.38)	0.83 (0.77, 0.88)	0.69 (0.62, 0.77)	1				
Lycopene	0.11 (-0.004, 0.16)	0.62 (0.56, 0.65)	0.26 (0.06, 0.36)	0.35 (0.25, 0.45)	1			
Lutein	0.24 (0.14, 0.30)	0.53 (0.50, 0.66)	0.39 (0.20, 0.52)	0.35 (0.33, 0.45)	0.25 (0.17, 0.38)	1		
Zeaxanthin	0.23 (0.11, 0.29)	0.43 (0.32, 0.46)	0.17 (0.05, 0.24)	0.22 (0.06, 0.28)	0.24 (0.05, 0.32)	0.46 (0.31, 0.54)	1	
β-cryptoxanthin	0.34 (0.30, 0.49)	0.62 (0.53, 0.74)	0.34 (0.21, 0.45)	0.44 (0.33, 0.51)	0.20 (0.13, 0.27)	0.29 (0.23, 0.44)	0.38 (0.34, 0.50)	1

* EPIC-InterAct subcohort participants was used to calculate the statistics across the eight countries in this table (n=12,589 for vitamin C [without measurements in Sweden]; and n=13,618 for carotenoids, in Spain, Italy, France, the UK, the Netherlands, Germany, Denmark, and Sweden);

† Pairwise Spearman correlation coefficients were calculated in each country. The average, minimum, and maximum are presented.

Supplemental Table S2 Associations of demographic, lifestyle and dietary factors with plasma vitamin C and carotenoids in the EPIC-InterAct Study*

	**Vitamin C**		**Total carotenoids**		**α-carotene**		**β-carotene**		**Lycopene**		**Lutein**		**Zeaxanthin,**		**β-cryptoxanthin**	
	Beta (95%CI)	I^2	Beta (95%CI)	I^2	Beta (95%CI)	I^2	Beta (95%CI)	I^2	Beta (95%CI)	I^2	Beta (95%CI)	I^2	Beta (95%CI)	I^2	Beta (95%CI)	I^2
**Demographic and lifestyle factors**																
Sex (women compared with men)	0.22 (0.14, 0.31)	47.2	0.07 (0.00, 0.14)	41.7	0.09 (0.02, 0.15)	37.2	0.14 (0.05, 0.23)	67.4	-0.10 (-0.19, 0.00)	62.1	0 (-0.05, 0.05)	0	-0.06 (-0.1, -0.02)	0	0.15 (0.10, 0.20)	0
Age (per 5y)	0.01 (0.00, 0.03)	7.2	0.01 (-0.02, 0.03)	76.5	0.02 (0.00, 0.03)	51.6	0.05 (0.04, 0.06)	0	-0.10 (-0.14, -0.06)	90.7	0.07 (0.06, 0.08)	0	0 (-0.01, 0.02)	43.9	0.02 (-0.01, 0.05)	85.6
BMI (per 5kg/m^2)	0.02 (-0.02, 0.06)	0	-0.09 (-0.16, -0.02)	72	-0.05 (-0.10, -0.01)	32.5	-0.08 (-0.14, -0.01)	70.7	-0.03 (-0.1, 0.03)	66.6	-0.13 (-0.16, -0.1)	0	-0.04 (-0.07, -0.01)	11.7	-0.02 (-0.07, 0.02)	45.8
Waist circumference (per 5cm)	-0.06 (-0.08, -0.04)	38.2	-0.07 (-0.10, -0.05)	52.4	-0.07 (-0.08, -0.05)	0	-0.09 (-0.11, -0.06)	65.8	-0.03 (-0.06, -0.01)	45	-0.03 (-0.05, -0.01)	0	-0.01 (-0.02, 0)	0	-0.05 (-0.06, -0.03)	0
Alcohol (per 15g/d)	0 (-0.02, 0.02)	31	-0.05 (-0.06, -0.03)	0	-0.05 (-0.07, -0.04)	6.1	-0.10 (-0.12, -0.07)	54.4	0.03 (-0.02, 0.08)	85.9	0 (-0.01, 0.02)	1.2	-0.02 (-0.04, 0.01)	62.4	-0.05 (-0.07, -0.03)	32
Season of blood draw (ref: winter), spring	-0.03 (-0.12, 0.07)	76.5	-0.05 (-0.11, 0.01)	56.1	-0.07 (-0.13, 0.00)	57.5	0 (-0.06, 0.06)	55	0.08 (0.02, 0.14)	47.9	0.13 (0.08, 0.18)	26.7	0.17 (0.09, 0.25)	76.8	-0.28 (-0.36, -0.19)	81
Season of blood draw (ref: winter), summer	0.01 (-0.14, 0.16)	88.3	0.02 (-0.04, 0.09)	47.5	0.03 (-0.11, 0.16)	88.6	0.18 (0.1, 0.26)	68.6	0.29 (0.16, 0.42)	84.3	0.12 (0.02, 0.23)	77.8	0.32 (0.17, 0.47)	91.1	-0.45 (-0.57, -0.34)	86.7
Season of blood draw (ref: winter), autumn	-0.05 (-0.18, 0.08)	85.9	-0.01 (-0.09, 0.07)	71.8	0.13 (0.08, 0.18)	25.3	0.17 (0.12, 0.21)	17.8	0.21 (0.12, 0.31)	78	0.04 (-0.03, 0.11)	64.9	0.05 (-0.03, 0.13)	73.6	-0.49 (-0.6, -0.37)	89.5
Smoking status (ref: never), former	-0.02 (-0.08, 0.03)	35.9	-0.05 (-0.09, -0.02)	0	-0.04 (-0.08, -0.01)	0	-0.07 (-0.1, -0.03)	0	0.01 (-0.03, 0.05)	0	-0.03 (-0.07, 0.01)	0	0.01 (-0.02, 0.04)	0	-0.07 (-0.10, -0.04)	0
Smoking status (ref: never), current	-0.17 (-0.22, -0.13)	0	-0.28 (-0.32, -0.25)	0	-0.31 (-0.40, -0.23)	75.4	-0.26 (-0.3, -0.23)	0	-0.06 (-0.12, -0.01)	33.1	-0.22 (-0.29, -0.15)	64.8	-0.08 (-0.12, -0.03)	31.2	-0.34 (-0.4, -0.29)	55.3
Physical activity (ref: inactive), moderate inactive	0.06 (0.00, 0.12)	27	0.03 (-0.02, 0.07)	18.6	0.02 (-0.04, 0.08)	38.8	0 (-0.06, 0.06)	46.8	0.05 (0.01, 0.09)	0	0.01 (-0.03, 0.05)	0	0.02 (-0.05, 0.08)	55.3	0.03 (0.00, 0.07)	0
Physical activity (ref: inactive), moderate active	0.09 (0.02, 0.15)	22.6	0.08 (0.04, 0.13)	0	0.08 (0.02, 0.14)	35.7	0.09 (0.04, 0.13)	0	0.06 (0.01, 0.11)	0	0.07 (0.03, 0.12)	0	0.01 (-0.02, 0.05)	0	0.04 (0.00, 0.08)	0
Physical activity (ref: inactive), active	0.13 (0.05, 0.21)	34.8	0.11 (0.06, 0.16)	0	0.07 (0.02, 0.12)	0	0.09 (0.04, 0.14)	0	0.07 (0.02, 0.13)	0	0.10 (0.05, 0.15)	0	0.06 (0.01, 0.1)	0	0.09 (0.04, 0.13)	0
Education level (ref: low), middle	0 (-0.06, 0.05)	35.9	0.06 (-0.01, 0.13)	67.5	0.09 (0.01, 0.17)	73.9	0.04 (-0.02, 0.1)	57.7	0.09 (-0.02, 0.2)	84.9	0.05 (-0.03, 0.13)	72.6	0.03 (0, 0.06)	0	0.01 (-0.03, 0.04)	0
Education level (ref: low), high	0.08 (0.00, 0.17)	53.1	0.15 (0.05, 0.25)	73.2	0.21 (0.11, 0.32)	77.4	0.16 (0.07, 0.24)	68	0.12 (-0.01, 0.25)	82.5	0.10 (0, 0.2)	72.2	0.04 (-0.05, 0.12)	67.8	0.08 (0.03, 0.12)	3
Employment (ref: no employment), yes	0.03 (-0.03, 0.08)	5.5	0.08 (0.04, 0.13)	0	0.05 (0.01, 0.09)	0	0.04 (-0.01, 0.08)	0	0.09 (0.04, 0.13)	0	0.07 (0, 0.15)	57	0.04 (0, 0.09)	0	0.10 (0.05, 0.15)	32.2
Marital status (ref: single), married	-0.06 (-0.17, 0.05)	26.3	0.02 (-0.08, 0.13)	52.2	0.01 (-0.1, 0.12)	60.1	0.03 (-0.07, 0.12)	40.2	0.08 (-0.01, 0.17)	30.5	0.03 (-0.05, 0.1)	4.9	0.03 (-0.07, 0.12)	39.1	0.03 (-0.06, 0.11)	39.1
Marital status (ref: single), separated/divorced	-0.05 (-0.23, 0.12)	40.4	-0.01 (-0.14, 0.12)	32.5	-0.04 (-0.14, 0.06)	0	-0.04 (-0.2, 0.12)	56.1	0.06 (-0.14, 0.26)	68.1	-0.08 (-0.19, 0.03)	0	0.04 (-0.06, 0.14)	0	0.02 (-0.11, 0.15)	45.6
Marital status (ref: single), widowed	-0.21 (-0.39, -0.02)	31.1	0.03 (-0.18, 0.23)	62.4	0 (-0.21, 0.22)	66.4	-0.03 (-0.22, 0.15)	53.2	0.15 (-0.04, 0.34)	51.1	-0.08 (-0.21, 0.05)	0	0.01 (-0.11, 0.14)	3.5	0.04 (-0.08, 0.15)	0
**Dietary factors**																
Total fruits and vegetables (per 100g/d)	0.08 (0.06, 0.10)	82.9	0.09 (0.07, 0.12)	86.9	0.10 (0.06, 0.09)	94.2	0.07 (0.06, 0.09)	71.1	0.03 (0.01, 0.04)	61.6	0.06 (0.04, 0.08)	81.6	0.02 (0.01, 0.03)	55.7	0.10 (0.08, 0.12)	87.7
Citrus fruits (per 100g/d)	0.19 (0.14, 0.25)	50.4	0.18 (0.14, 0.22)	28.7	0.02 (-0.03, 0.07)	51.9	0.05 (0.03, 0.08)	0	0.05 (0.01, 0.08)	17	0.02 (0, 0.04)	0	0.16 (0.1, 0.23)	74.4	0.51 (0.38, 0.65)	95.6
Non-citrus fruits (per 100g/d)	0.05 (0.03, 0.06)	0	0.03 (0.01, 0.05)	34.5	0.06 (0.03, 0.08)	48.3	0.04 (0.03, 0.05)	0	-0.01 (-0.04, 0.01)	56.9	0.02 (0.01, 0.04)	7.5	-0.03 (-0.04, -0.01)	33.6	0.04 (0.02, 0.06)	63.4

	Vitamin C		Total carotenoids		α-carotene		β-carotene		Lycopene		Lutein		Zeaxanthin,		β-cryptoxanthin	
	Beta (95%CI)	I^2	Beta (95%CI)	I^2	Beta (95%CI)	I^2	Beta (95%CI)	I^2	Beta (95%CI)	I^2	Beta (95%CI)	I^2	Beta (95%CI)	I^2	Beta (95%CI)	I^2
Green leafy vegetables (per 100g/d)	0.04 (-0.01, 0.09)	0	0.01 (-0.04, 0.06)	0	-0.07 (-0.21, 0.06)	47.8	0.09 (0.02, 0.17)	9.5	-0.12 (-0.26, 0.01)	43.6	0.34 (0.14, 0.54)	72.5	0.08 (-0.1, 0.25)	66.1	-0.03 (-0.07, 0.01)	0
Fruiting vegetables (per 100g/d)	0.12 (0.04, 0.21)	57.2	0.21 (0.09, 0.34)	85.8	0.05 (-0.05, 0.14)	75	0.14 (0.04, 0.23)	76.8	0.38 (0.22, 0.55)	91.8	0.11 (0.03, 0.18)	57.5	0.07 (0.01, 0.12)	37.9	0.1 (0.04, 0.17)	55.3
Root vegetables (per 100g/d)	0.10 (-0.01, 0.20)	21.1	0.39 (0.28, 0.50)	44.4	1.20 (0.95, 1.45)	90.7	0.50 (0.38, 0.63)	60.2	-0.07 (-0.18, 0.03)	39.5	0.10 (0.03, 0.17)	0	-0.09 (-0.18, 0.00)	34.6	-0.01 (-0.08, 0.05)	0
Cabbage (per 100g/d)	0.12 (-0.01, 0.24)	29	0.10 (-0.07, 0.26)	64.3	0.20 (0.00, 0.39)	76.4	0.15 (0.01, 0.29)	51.8	-0.03 (-0.20, 0.13)	55.1	0.25 (0.05, 0.44)	72.8	0 (-0.12, 0.11)	32.4	0.01 (-0.13, 0.16)	62.9
Other vegetables (per 100g/d)	0.01 (-0.07, 0.09)	56.9	0.05 (-0.04, 0.14)	71.9	-0.01 (-0.11, 0.1)	78.6	0.03 (-0.05, 0.11)	60.2	0.08 (-0.03, 0.20)	79	0.14 (0.09, 0.19)	18	0.03 (-0.06, 0.12)	68.8	0.03 (-0.02, 0.08)	25.8
Potatoes (per 100g/d)	0.03 (-0.01, 0.07)	25.2	0.02 (-0.03, 0.07)	62.8	0.02 (-0.04, 0.07)	74.8	0 (-0.05, 0.04)	61	0.03 (-0.03, 0.09)	73.8	0.03 (-0.02, 0.09)	71.2	-0.01 (-0.04, 0.02)	18.7	-0.01 (-0.04, 0.03)	47.2
Cereal and cereal products (per 200g/d)	0.03 (-0.01, 0.08)	0	0.19 (0.09, 0.29)	80.7	0.16 (0.08, 0.25)	72.7	0.11 (0.04, 0.18)	62.6	0.25 (0.16, 0.34)	72.8	0.15 (0.07, 0.23)	69.1	-0.04 (-0.09, 0.02)	41.3	0.01 (-0.03, 0.06)	19.2
Milk and dairy products (per 200g/d)	-0.01 (-0.04, 0.01)	41.2	0.01 (-0.01, 0.03)	44.2	0.03 (0.02, 0.05)	0	0.03 (0.01, 0.05)	29.4	0.01 (-0.02, 0.03)	56.4	-0.02 (-0.04, -0.01)	0	-0.02 (-0.04, 0.01)	50.9	0 (-0.02, 0.01)	40.3
Fruit and vegetable juice (per 200g/d)	0.28 (0.14, 0.41)	90.5	0.14 (0.11, 0.18)	9.6	0.08 (0.02, 0.14)	60.7	0.09 (0.04, 0.14)	41.6	0.04 (0.00, 0.09)	14.3	0.08 (0.02, 0.15)	64.3	0.28 (0.15, 0.42)	93.7	0.24 (0.14, 0.34)	90.3
Soft drink (per 200g/d)	0 (-0.04, 0.03)	51.3	-0.02 (-0.05, 0.01)	50	-0.05 (-0.08, -0.02)	48	-0.02 (-0.06, 0.01)	62.1	0 (-0.02, 0.02)	0	-0.04 (-0.06, -0.02)	0	-0.02 (-0.04, 0.00)	0	-0.02 (-0.04, 0.01)	17.9
Fish (per 100g/d)	0.15 (0.02, 0.28)	64.2	0.13 (0.00, 0.26)	73.2	0.07 (-0.02, 0.15)	39.9	0.07 (-0.04, 0.18)	61.2	0.09 (-0.05, 0.23)	72	0.09 (-0.03, 0.21)	66.8	0.05 (-0.07, 0.17)	64.8	0 (-0.08, 0.08)	41.4
Red meat (per 100g/d)	-0.09 (-0.17, -0.01)	35.4	0.05 (-0.05, 0.15)	61.5	-0.02 (-0.12, 0.07)	57.3	-0.01 (-0.1, 0.08)	55.1	0.16 (0.05, 0.27)	64.9	-0.06 (-0.12, 0.01)	14.1	-0.01 (-0.08, 0.06)	28.5	0.01 (-0.04, 0.06)	0.8
Legumes (per 50g/d)	0 (-0.13, 0.13)	52.9	0.03 (-0.09, 0.16)	62.7	0.02 (-0.02, 0.06)	0	0 (-0.04, 0.03)	0	0.04 (-0.18, 0.27)	86.4	0 (-0.12, 0.13)	59.3	-0.07 (-0.22, 0.08)	66.2	-0.06 (-0.18, 0.05)	61.9
Egg and egg products (per 50g/d)	-0.08 (-0.14, -0.03)	0	-0.02 (-0.07, 0.03)	0	-0.07 (-0.13, 0.00)	28.6	-0.05 (-0.10, 0.00)	0	-0.05 (-0.10, 0.00)	0	0.11 (0.00, 0.23)	69.3	0.16 (0.12, 0.20)	0	-0.06 (-0.13, 0)	38.2
Nuts and seeds (per 10g/d)	0.02 (-0.02, 0.06)	41.8	0.05 (0.00, 0.10)	66.4	0.03 (0.00, 0.06)	41.3	0.02 (-0.02, 0.06)	57.8	0.06 (-0.01, 0.13)	79.5	0.03 (-0.01, 0.07)	50.8	0.03 (-0.02, 0.07)	61.1	0.03 (0, 0.07)	50.8
Offals (per 10g/d)	0.03 (0.00, 0.06)	0	-0.01 (-0.04, 0.03)	10.4	0 (-0.03, 0.03)	0	0 (-0.03, 0.03)	0	0 (-0.04, 0.04)	11.8	-0.03 (-0.09, 0.03)	61.3	-0.01 (-0.05, 0.03)	21.6	0 (-0.05, 0.04)	44.3
Vitamin supplements (yes vs no)	0.27 (0.08, 0.47)	95.9	0.05 (-0.03, 0.12)	77.6	0.06 (0.03, 0.09)	0	0.09 (0.01, 0.18)	84.1	-0.05 (-0.09, -0.02)	0	-0.04 (-0.07, 0.00)	0	0.02 (-0.04, 0.08)	72.5	0.01 (-0.04, 0.05)	54.8

* The betas (95%CI) in the table represent differences in vitamin C and total carotenoids (in SD unit) per 1-standardised unit/category difference in each demographic, lifestyle or dietary factor. Linear regression was used to obtain the country-specific estimate of an association, which was combined across countries using random effects meta-analysis. Covariates in the model included all the variables in this table. Sample size was 10,584 for vitamin C and 11,537 for the carotenoids estimation. All of the carotenoids variables were natural log-transformed before statistical analysis in the linear regression. SD was 19.2 µmol/L for vitamin C, 0.50 for total carotenoids (ln-transformed), 0.91 for α-carotene (ln-transformed), 0.73 for β-carotene (ln-transformed), 0.68 for lycopene (ln-transformed), 0.51 for lutein (ln-transformed), 0.91 for zeaxanthin (ln-transformed) and 0.94 for β-cryptoxanthin (ln-transformed).

Supplemental Table S3 Sensitivity analysis for the association between plasma vitamin C and carotenoids and type 2 diabetes in the EPIC-InterAct Study

	Models	N	Hazard ratios (95% Confidence Intervals)*					
			Q1	Q2	Q3	Q4	Q5	Per SD
Vitamin C								
	Model 2†	19,255	1.0 (ref)	0.94 (0.83, 1.08)	0.72 (0.60, 0.87)	0.68 (0.56, 0.82)	0.58 (0.47, 0.72)	0.82 (0.76, 0.89)
	Model 2, with multiple imputation	21,018	1.0 (ref)	1.00 (0.88, 1.13)	0.73 (0.62, 0.86)	0.70 (0.58, 0.85)	0.62 (0.51, 0.76)	0.84 (0.78, 0.91)
	Model 2, excluding HbA1c ≥ 6.5%	17,629	1.0 (ref)	0.99 (0.88, 1.11)	0.79 (0.67, 0.93)	0.77 (0.65, 0.91)	0.69 (0.58, 0.81)	0.87 (0.82, 0.93)
	Model 2, excluding first 2 years' incident T2D	18,445	1.0 (ref)	0.96 (0.84, 1.10)	0.75 (0.62, 0.91)	0.71 (0.58, 0.85)	0.60 (0.50, 0.73)	0.84 (0.78, 0.90)
	Model 2, excluding first 4 years' incident T2D	17,295	1.0 (ref)	0.96 (0.82, 1.11)	0.78 (0.64, 0.95)	0.72 (0.58, 0.89)	0.61 (0.50, 0.75)	0.85 (0.79, 0.92)
	Model 2, restricting to first 8 years' follow up	19,256	1.0 (ref)	0.98 (0.84, 1.14)	0.73 (0.62, 0.87)	0.68 (0.56, 0.82)	0.53 (0.42, 0.67)	0.80 (0.74, 0.87)
	Model 2, excluding baseline cancer and CVD cases	18,294	1.0 (ref)	0.97 (0.84, 1.12)	0.73 (0.59, 0.90)	0.68 (0.55, 0.84)	0.59 (0.48, 0.73)	0.82 (0.76, 0.89)
	Model 2, + plasma individual carotenoids	19,206	1.0 (ref)	1.00 (0.90, 1.12)	0.80 (0.68, 0.95)	0.78 (0.68, 0.89)	0.69 (0.57, 0.83)	0.88 (0.82, 0.94)
	Model 2, + vitamin supplements	17,425	1.0 (ref)	0.95 (0.82, 1.10)	0.73 (0.60, 0.88)	0.70 (0.57, 0.86)	0.60 (0.49, 0.75)	0.83 (0.77, 0.91)
	Model 2, + season of blood draw	19,211	1.0 (ref)	0.95 (0.83, 1.07)	0.71 (0.59, 0.86)	0.67 (0.55, 0.81)	0.56 (0.45, 0.71)	0.81 (0.75, 0.89)
	Model 2, + family history and disease prevalence‡	16,263	1.0 (ref)	0.95 (0.78, 1.16)	0.71 (0.56, 0.91)	0.71 (0.56, 0.90)	0.62 (0.49, 0.78)	0.84 (0.78, 0.91)
	Model 2, + hormone use and menopause status in women	19,255	1.0 (ref)	0.94 (0.82, 1.08)	0.72 (0.59, 0.87)	0.68 (0.56, 0.83)	0.58 (0.47, 0.72)	0.82 (0.76, 0.89)
	Model 2, + BMI-GRS and IR-GRS§	15525	1.0 (ref)	0.91 (0.79, 1.04)	0.71 (0.61, 0.83)	0.69 (0.56, 0.85)	0.56 (0.45, 0.71)	0.82 (0.75, 0.89)
	Model 2, + diet quality score§	19256	1.0 (ref)	0.94 (0.81, 1.09)	0.72 (0.60, 0.87)	0.68 (0.56, 0.83)	0.59 (0.48, 0.73)	0.83 (0.76, 0.90)
Total carotenoids								
	Model 2†	19,907	1.0 (ref)	0.83 (0.74, 0.92)	0.64 (0.54, 0.75)	0.61 (0.53, 0.70)	0.51 (0.43, 0.60)	0.75 (0.68, 0.82)
	Model 2, with multiple imputation	22,742	1.0 (ref)	0.81 (0.73, 0.89)	0.64 (0.56, 0.72)	0.60 (0.52, 0.70)	0.49 (0.41, 0.59)	0.74 (0.67, 0.81)
	Model 2, excluding HbA1c ≥ 6.5%	18,319	1.0 (ref)	0.88 (0.79, 0.98)	0.67 (0.57, 0.77)	0.63 (0.55, 0.72)	0.55 (0.46, 0.67)	0.77 (0.70, 0.85)
	Model 2, excluding first 2 years' incident T2D	19,188	1.0 (ref)	0.84 (0.76, 0.94)	0.65 (0.54, 0.77)	0.63 (0.55, 0.72)	0.54 (0.46, 0.63)	0.76 (0.70, 0.83)
	Model 2, excluding first 4 years' incident T2D	18,102	1.0 (ref)	0.87 (0.78, 0.97)	0.66 (0.55, 0.79)	0.65 (0.56, 0.77)	0.54 (0.45, 0.64)	0.77 (0.71, 0.84)
	Model 2, restricting to first 8 years' follow up	19,909	1.0 (ref)	0.83 (0.74, 0.94)	0.61 (0.52, 0.73)	0.60 (0.51, 0.70)	0.49 (0.41, 0.59)	0.73 (0.66, 0.81)
	Model 2, excluding baseline cancer and CVD cases	17,535	1.0 (ref)	0.84 (0.75, 0.94)	0.66 (0.58, 0.75)	0.58 (0.50, 0.67)	0.48 (0.40, 0.58)	0.74 (0.66, 0.82)
	Model 2, + plasma vitamin C	19,746	1.0 (ref)	0.86 (0.77, 0.95)	0.67 (0.57, 0.80)	0.65 (0.57, 0.74)	0.55 (0.47, 0.65)	0.78 (0.73, 0.84)
	Model 2, + vitamin supplements	18,315	1.0 (ref)	0.82 (0.73, 0.91)	0.64 (0.55, 0.75)	0.61 (0.53, 0.70)	0.53 (0.45, 0.62)	0.76 (0.69, 0.83)
	Model 2, + season of blood draw	19,866	1.0 (ref)	0.82 (0.74, 0.91)	0.63 (0.53, 0.74)	0.60 (0.53, 0.69)	0.51 (0.43, 0.60)	0.75 (0.68, 0.82)
	Model 2, + family history and disease prevalence‡	17,183	1.0 (ref)	0.88 (0.78, 1.00)	0.75 (0.62, 0.92)	0.64 (0.53, 0.78)	0.58 (0.49, 0.70)	0.78 (0.71, 0.86)
	Model 2, + hormone use and menopause status in women	19,907	1.0 (ref)	0.82 (0.74, 0.92)	0.63 (0.54, 0.75)	0.61 (0.52, 0.71)	0.51 (0.42, 0.61)	0.74 (0.67, 0.82)
	Model 2, + BMI-GRS and IR-GRS§	16162	1.0 (ref)	0.80 (0.67, 0.96)	0.63 (0.52, 0.77)	0.57 (0.48, 0.66)	0.49 (0.39, 0.61)	0.76 (0.71, 0.81)
	Model 2, + diet quality score§	19909	1.0 (ref)	0.83 (0.75, 0.92)	0.64 (0.54, 0.75)	0.61 (0.53, 0.70)	0.51 (0.43, 0.60)	0.75 (0.69, 0.83)
α-carotene								
	Model 2†	19,907	1.0 (ref)	0.81 (0.70, 0.94)	0.72 (0.64, 0.82)	0.69 (0.59, 0.79)	0.53 (0.46, 0.62)	0.79 (0.72, 0.88)
	Model 2, with multiple imputation	22,742	1.0 (ref)	0.78 (0.66, 0.93)	0.70 (0.62, 0.79)	0.64 (0.55, 0.73)	0.52 (0.46, 0.60)	0.80 (0.74, 0.87)

	Models	N	Hazard ratios (95% Confidence Intervals)* Q1	Q2	Q3	Q4	Q5	Per SD
	Model 2, excluding HbA1c ≥ 6.5%	18,319	1.0 (ref)	0.84 (0.72, 0.98)	0.74 (0.66, 0.83)	0.73 (0.61, 0.87)	0.56 (0.48, 0.65)	0.81 (0.73, 0.90)
	Model 2, excluding first 2 years' incident T2D	19,188	1.0 (ref)	0.83 (0.71, 0.97)	0.72 (0.64, 0.80)	0.70 (0.60, 0.81)	0.55 (0.47, 0.63)	0.80 (0.72, 0.89)
	Model 2, excluding first 4 years' incident T2D	18,102	1.0 (ref)	0.85 (0.71, 1.02)	0.72 (0.64, 0.82)	0.72 (0.60, 0.87)	0.55 (0.47, 0.64)	0.80 (0.72, 0.90)
	Model 2, restricting to first 8 years' follow up	19,909	1.0 (ref)	0.82 (0.69, 0.98)	0.75 (0.66, 0.85)	0.68 (0.59, 0.79)	0.56 (0.46, 0.69)	0.81 (0.73, 0.90)
	Model 2, excluding baseline cancer and CVD cases	17,535	1.0 (ref)	0.84 (0.72, 0.98)	0.74 (0.63, 0.86)	0.72 (0.61, 0.84)	0.51 (0.43, 0.62)	0.76 (0.67, 0.86)
	Model 2, + plasma vitamin C, individual carotenoids	19,746	1.0 (ref)	0.87 (0.75, 1.01)	0.82 (0.72, 0.95)	0.85 (0.74, 0.99)	0.78 (0.66, 0.92)	0.96 (0.90, 1.03)
	Model 2, + vitamin supplements	18,315	1.0 (ref)	0.79 (0.67, 0.94)	0.71 (0.63, 0.80)	0.66 (0.58, 0.75)	0.52 (0.44, 0.61)	0.79 (0.71, 0.88)
	Model 2, + season of blood draw	19,866	1.0 (ref)	0.80 (0.69, 0.94)	0.72 (0.64, 0.80)	0.67 (0.58, 0.77)	0.52 (0.44, 0.62)	0.79 (0.71, 0.88)
	Model 2, + family history and disease prevalence‡	17,183	1.0 (ref)	0.76 (0.60, 0.96)	0.67 (0.55, 0.82)	0.66 (0.53, 0.82)	0.50 (0.42, 0.59)	0.79 (0.72, 0.88)
	Model 2, + hormone use and menopause status in women	19,907	1.0 (ref)	0.81 (0.70, 0.94)	0.71 (0.63, 0.80)	0.68 (0.59, 0.78)	0.53 (0.45, 0.62)	0.79 (0.71, 0.89)
	Model 2, + BMI-GRS and IR-GRS§	16162	1.0 (ref)	0.79 (0.70, 0.90)	0.73 (0.65, 0.83)	0.66 (0.57, 0.76)	0.51 (0.44, 0.60)	0.79 (0.70, 0.89)
	Model 2, + diet quality score§	19909	1.0 (ref)	0.81 (0.70, 0.94)	0.73 (0.65, 0.81)	0.69 (0.59, 0.80)	0.54 (0.47, 0.62)	0.80 (0.72, 0.89)
β-carotene								
	Model 2†	19,907	1.0 (ref)	0.78 (0.69, 0.88)	0.60 (0.54, 0.68)	0.53 (0.47, 0.60)	0.45 (0.39, 0.52)	0.71 (0.62, 0.82)
	Model 2, with multiple imputation	22,742	1.0 (ref)	0.74 (0.66, 0.83)	0.61 (0.54, 0.68)	0.50 (0.45, 0.57)	0.43 (0.37, 0.50)	0.70 (0.61, 0.80)
	Model 2, excluding HbA1c ≥ 6.5%	18,319	1.0 (ref)	0.80 (0.68, 0.94)	0.63 (0.56, 0.71)	0.57 (0.50, 0.65)	0.48 (0.41, 0.58)	0.75 (0.65, 0.85)
	Model 2, excluding first 2 years' incident T2D	19,188	1.0 (ref)	0.79 (0.68, 0.91)	0.61 (0.54, 0.69)	0.54 (0.47, 0.62)	0.46 (0.39, 0.53)	0.72 (0.63, 0.82)
	Model 2, excluding first 4 years' incident T2D	18,102	1.0 (ref)	0.80 (0.68, 0.95)	0.61 (0.54, 0.69)	0.56 (0.49, 0.64)	0.45 (0.36, 0.56)	0.73 (0.64, 0.85)
	Model 2, restricting to first 8 years' follow up	19,909	1.0 (ref)	0.75 (0.66, 0.87)	0.59 (0.52, 0.68)	0.50 (0.43, 0.58)	0.46 (0.39, 0.54)	0.69 (0.60, 0.79)
	Model 2, excluding baseline cancer and CVD cases	17,535	1.0 (ref)	0.78 (0.69, 0.87)	0.60 (0.53, 0.67)	0.53 (0.46, 0.61)	0.43 (0.35, 0.51)	0.69 (0.58, 0.83)
	Model 2, + plasma vitamin C, individual carotenoids	19,746	1.0 (ref)	0.80 (0.68, 0.94)	0.65 (0.57, 0.73)	0.57 (0.49, 0.66)	0.50 (0.40, 0.63)	0.78 (0.69, 0.89)
	Model 2, + vitamin supplements	18,315	1.0 (ref)	0.78 (0.66, 0.92)	0.59 (0.53, 0.67)	0.53 (0.46, 0.60)	0.46 (0.39, 0.54)	0.73 (0.62, 0.84)
	Model 2, + season of blood draw	19,866	1.0 (ref)	0.77 (0.68, 0.88)	0.59 (0.52, 0.67)	0.52 (0.45, 0.59)	0.43 (0.37, 0.51)	0.70 (0.60, 0.82)
	Model 2, + family history and disease prevalence‡	17,183	1.0 (ref)	0.73 (0.64, 0.82)	0.62 (0.53, 0.72)	0.53 (0.46, 0.61)	0.48 (0.40, 0.56)	0.75 (0.63, 0.89)
	Model 2, + hormone use and menopause status in women	19,907	1.0 (ref)	0.76 (0.68, 0.85)	0.60 (0.53, 0.67)	0.52 (0.46, 0.59)	0.43 (0.37, 0.50)	0.70 (0.61, 0.81)
	Model 2, + BMI-GRS and IR-GRS§	16162	1.0 (ref)	0.81 (0.70, 0.93)	0.60 (0.53, 0.69)	0.54 (0.46, 0.62)	0.45 (0.38, 0.53)	0.70 (0.61, 0.81)
	Model 2, + diet quality score§	19909	1.0 (ref)	0.78 (0.68, 0.89)	0.60 (0.54, 0.68)	0.53 (0.47, 0.61)	0.45 (0.39, 0.52)	0.71 (0.62, 0.82)
Lycopene								
	Model 2†	19,907	1.0 (ref)	0.95 (0.86, 1.06)	0.84 (0.75, 0.94)	0.81 (0.71, 0.92)	0.79 (0.68, 0.93)	0.91 (0.85, 0.98)
	Model 2, with multiple imputation	22,742	1.0 (ref)	0.96 (0.86, 1.06)	0.83 (0.75, 0.93)	0.80 (0.71, 0.90)	0.76 (0.64, 0.90)	0.90 (0.84, 0.97)
	Model 2, excluding HbA1c ≥ 6.5%	18,319	1.0 (ref)	0.91 (0.77, 1.09)	0.87 (0.77, 0.97)	0.83 (0.73, 0.95)	0.81 (0.68, 0.98)	0.93 (0.87, 0.99)
	Model 2, excluding first 2 years' incident T2D	19,188	1.0 (ref)	0.96 (0.86, 1.07)	0.85 (0.76, 0.96)	0.83 (0.73, 0.94)	0.83 (0.70, 0.97)	0.93 (0.86, 0.99)
	Model 2, excluding first 4 years' incident T2D	18,102	1.0 (ref)	0.97 (0.85, 1.11)	0.90 (0.78, 1.03)	0.84 (0.73, 0.96)	0.83 (0.68, 1.01)	0.93 (0.87, 1.00)
	Model 2, restricting to first 8 years' follow up	19,909	1.0 (ref)	0.93 (0.82, 1.05)	0.80 (0.69, 0.94)	0.82 (0.71, 0.94)	0.80 (0.68, 0.95)	0.92 (0.85, 0.99)
	Model 2, excluding baseline cancer and CVD cases	17,535	1.0 (ref)	0.99 (0.88, 1.11)	0.84 (0.74, 0.95)	0.84 (0.74, 0.97)	0.82 (0.70, 0.97)	0.93 (0.86, 0.99)
	Model 2, + plasma vitamin C, individual carotenoids	19,746	1.0 (ref)	1.01 (0.91, 1.13)	0.92 (0.82, 1.04)	0.93 (0.81, 1.06)	0.96 (0.79, 1.16)	0.98 (0.91, 1.06)
	Model 2, + vitamin supplements	18,315	1.0 (ref)	0.96 (0.85, 1.07)	0.84 (0.75, 0.95)	0.81 (0.71, 0.92)	0.81 (0.70, 0.94)	0.92 (0.86, 0.98)

	Models	N	Hazard ratios (95% Confidence Intervals)*					
			Q1	Q2	Q3	Q4	Q5	Per SD
	Model 2, + season of blood draw	19,866	1.0 (ref)	0.95 (0.85, 1.06)	0.83 (0.74, 0.93)	0.79 (0.70, 0.90)	0.77 (0.65, 0.91)	0.91 (0.84, 0.97)
	Model 2, + family history and disease prevalence‡	15,745	1.0 (ref)	0.91 (0.78, 1.07)	0.85 (0.75, 0.96)	0.80 (0.69, 0.93)	0.86 (0.74, 1.01)	0.94 (0.88, 1.02)
	Model 2, + hormone use and menopause status in women	19,907	1.0 (ref)	0.95 (0.85, 1.06)	0.84 (0.74, 0.94)	0.81 (0.71, 0.91)	0.8 (0.69, 0.93)	0.91 (0.85, 0.98)
	Model 2, + BMI-GRS and IR-GRS§	16162	1.0 (ref)	0.98 (0.87, 1.11)	0.87 (0.77, 0.99)	0.81 (0.70, 0.93)	0.78 (0.63, 0.96)	0.92 (0.86, 0.98)
	Model 2, + diet quality score§	19909	1.0 (ref)	0.96 (0.86, 1.07)	0.84 (0.75, 0.95)	0.82 (0.72, 0.93)	0.81 (0.70, 0.94)	0.92 (0.86, 0.98)
Lutein								
	Model 2†	19,907	1.0 (ref)	0.89 (0.78, 1.01)	0.75 (0.65, 0.86)	0.70 (0.59, 0.82)	0.65 (0.55, 0.78)	0.84 (0.77, 0.91)
	Model 2, with multiple imputation	22,742	1.0 (ref)	0.84 (0.75, 0.93)	0.73 (0.64, 0.83)	0.70 (0.61, 0.80)	0.65 (0.56, 0.75)	0.83 (0.77, 0.90)
	Model 2, excluding HbA1c ≥ 6.5%	18,319	1.0 (ref)	0.91 (0.80, 1.04)	0.79 (0.70, 0.89)	0.76 (0.67, 0.87)	0.67 (0.57, 0.79)	0.86 (0.80, 0.93)
	Model 2, excluding first 2 years' incident T2D	19,188	1.0 (ref)	0.89 (0.77, 1.02)	0.78 (0.68, 0.90)	0.71 (0.59, 0.85)	0.66 (0.54, 0.80)	0.85 (0.79, 0.92)
	Model 2, excluding first 4 years' incident T2D	18,102	1.0 (ref)	0.90 (0.78, 1.04)	0.81 (0.71, 0.91)	0.73 (0.61, 0.86)	0.64 (0.54, 0.76)	0.86 (0.81, 0.91)
	Model 2, restricting to first 8 years' follow up	19,909	1.0 (ref)	0.90 (0.74, 1.09)	0.69 (0.56, 0.85)	0.61 (0.48, 0.77)	0.64 (0.50, 0.83)	0.81 (0.72, 0.90)
	Model 2, excluding baseline cancer and CVD cases	17,535	1.0 (ref)	0.88 (0.77, 1.01)	0.76 (0.67, 0.86)	0.72 (0.59, 0.87)	0.65 (0.55, 0.77)	0.84 (0.77, 0.91)
	Model 2, + plasma vitamin C, individual carotenoids	19,746	1.0 (ref)	0.94 (0.80, 1.11)	0.80 (0.68, 0.95)	0.77 (0.60, 0.98)	0.75 (0.57, 1.00)	0.91 (0.83, 0.99)
	Model 2, + vitamin supplements	18,315	1.0 (ref)	0.88 (0.77, 1.01)	0.76 (0.67, 0.86)	0.70 (0.59, 0.82)	0.63 (0.54, 0.75)	0.82 (0.75, 0.90)
	Model 2, + season of blood draw	19,866	1.0 (ref)	0.88 (0.77, 1.01)	0.73 (0.64, 0.85)	0.67 (0.56, 0.80)	0.63 (0.52, 0.77)	0.82 (0.75, 0.90)
	Model 2, + family history and disease prevalence‡	17,183	1.0 (ref)	0.88 (0.77, 1.01)	0.76 (0.66, 0.88)	0.71 (0.58, 0.88)	0.62 (0.52, 0.74)	0.83 (0.76, 0.91)
	Model 2, + hormone use and menopause status in women	19,907	1.0 (ref)	0.89 (0.77, 1.03)	0.74 (0.64, 0.85)	0.68 (0.56, 0.81)	0.65 (0.54, 0.78)	0.83 (0.76, 0.91)
	Model 2, + BMI-GRS and IR-GRS§	16162	1.0 (ref)	0.86 (0.71, 1.04)	0.74 (0.59, 0.93)	0.72 (0.61, 0.85)	0.64 (0.51, 0.81)	0.86 (0.79, 0.93)
	Model 2, + diet quality score§	19909	1.0 (ref)	0.89 (0.78, 1.02)	0.76 (0.67, 0.86)	0.70 (0.60, 0.83)	0.65 (0.55, 0.77)	0.84 (0.78, 0.91)
Zeaxanthin								
	Model 2†	19,907	1.0 (ref)	0.95 (0.84, 1.07)	0.98 (0.86, 1.13)	0.99 (0.78, 1.27)	0.86 (0.66, 1.12)	0.96 (0.88, 1.05)
	Model 2, with multiple imputation	22,742	1.0 (ref)	0.99 (0.88, 1.10)	1.01 (0.89, 1.14)	1.01 (0.83, 1.25)	0.90 (0.69, 1.18)	0.97 (0.89, 1.05)
	Model 2, excluding HbA1c ≥ 6.5%	18,319	1.0 (ref)	0.94 (0.83, 1.06)	0.95 (0.83, 1.10)	0.99 (0.78, 1.27)	0.77 (0.62, 0.96)	0.95 (0.88, 1.03)
	Model 2, excluding first 2 years' incident T2D	19,188	1.0 (ref)	0.95 (0.84, 1.07)	0.99 (0.86, 1.14)	1.01 (0.79, 1.28)	0.84 (0.67, 1.05)	0.97 (0.88, 1.06)
	Model 2, excluding first 4 years' incident T2D	18,102	1.0 (ref)	0.97 (0.85, 1.10)	1.04 (0.90, 1.20)	1.04 (0.81, 1.34)	0.90 (0.68, 1.20)	0.99 (0.89, 1.09)
	Model 2, restricting to first 8 years' follow up	19,909	1.0 (ref)	0.94 (0.80, 1.10)	0.93 (0.80, 1.09)	0.96 (0.76, 1.22)	0.83 (0.65, 1.06)	0.95 (0.85, 1.05)
	Model 2, excluding baseline cancer and CVD cases	17,535	1.0 (ref)	0.93 (0.81, 1.06)	0.98 (0.82, 1.18)	0.93 (0.71, 1.22)	0.78 (0.57, 1.05)	0.94 (0.85, 1.04)
	Model 2, + plasma vitamin C, individual carotenoids	19,746	1.0 (ref)	1.04 (0.92, 1.18)	1.17 (1.00, 1.37)	1.24 (0.95, 1.62)	1.12 (0.81, 1.55)	1.10 (0.97, 1.25)
	Model 2, + vitamin supplements	18,315	1.0 (ref)	0.97 (0.85, 1.10)	1.01 (0.87, 1.18)	1.05 (0.80, 1.38)	0.88 (0.67, 1.16)	0.98 (0.90, 1.07)
	Model 2, + season of blood draw	19,866	1.0 (ref)	0.94 (0.82, 1.09)	0.97 (0.84, 1.11)	0.96 (0.74, 1.25)	0.83 (0.64, 1.09)	0.95 (0.87, 1.04)
	Model 2, + family history and disease prevalence‡	17,183	1.0 (ref)	0.96 (0.80, 1.15)	1.02 (0.84, 1.23)	1.06 (0.84, 1.35)	0.98 (0.70, 1.37)	1.00 (0.90, 1.10)
	Model 2, + hormone use and menopause status in women	19,907	1.0 (ref)	0.95 (0.84, 1.08)	1.00 (0.87, 1.16)	1.01 (0.78, 1.30)	0.87 (0.66, 1.14)	0.96 (0.87, 1.06)
	Model 2, + BMI-GRS and IR-GRS§	16162	1.0 (ref)	0.92 (0.81, 1.05)	0.94 (0.81, 1.10)	0.99 (0.82, 1.20)	0.84 (0.64, 1.10)	0.97 (0.88, 1.08)
	Model 2, + diet quality score§	19909	1.0 (ref)	0.96 (0.85, 1.08)	0.99 (0.86, 1.13)	1.01 (0.79, 1.28)	0.85 (0.65, 1.10)	0.96 (0.88, 1.05)
β-cryptoxanthin								
	Model 2†	19,907	1.0 (ref)	0.81 (0.68, 0.97)	0.81 (0.69, 0.95)	0.83 (0.72, 0.96)	0.72 (0.56, 0.93)	0.88 (0.81, 0.96)

Models	N	Hazard ratios (95% Confidence Intervals)*					
		Q1	Q2	Q3	Q4	Q5	Per SD
Model 2, with multiple imputation	22,742	1.0 (ref)	0.83 (0.74, 0.95)	0.77 (0.64, 0.94)	0.82 (0.71, 0.93)	0.73 (0.57, 0.94)	0.86 (0.79, 0.94)
Model 2, excluding HbA1c ≥ 6.5%	18,319	1.0 (ref)	0.85 (0.69, 1.05)	0.83 (0.72, 0.97)	0.86 (0.71, 1.04)	0.76 (0.57, 1.00)	0.88 (0.81, 0.96)
Model 2, excluding first 2 years' incident T2D	19,188	1.0 (ref)	0.83 (0.69, 0.99)	0.82 (0.71, 0.95)	0.86 (0.74, 0.99)	0.75 (0.59, 0.97)	0.89 (0.82, 0.96)
Model 2, excluding first 4 years' incident T2D	18,102	1.0 (ref)	0.82 (0.67, 0.99)	0.83 (0.71, 0.98)	0.86 (0.71, 1.03)	0.75 (0.57, 0.98)	0.90 (0.83, 0.97)
Model 2, restricting to first 8 years' follow up	19,909	1.0 (ref)	0.81 (0.67, 0.98)	0.83 (0.68, 1.01)	0.80 (0.67, 0.95)	0.70 (0.53, 0.93)	0.86 (0.78, 0.94)
Model 2, excluding baseline cancer and CVD cases	17,535	1.0 (ref)	0.78 (0.62, 0.98)	0.77 (0.61, 0.98)	0.82 (0.68, 0.98)	0.63 (0.47, 0.86)	0.86 (0.78, 0.96)
Model 2, + plasma vitamin C, individual carotenoids	19,746	1.0 (ref)	0.92 (0.78, 1.08)	0.98 (0.85, 1.13)	1.04 (0.89, 1.21)	0.99 (0.82, 1.19)	0.98 (0.92, 1.03)
Model 2, + vitamin supplements	18,315	1.0 (ref)	0.82 (0.68, 0.99)	0.80 (0.67, 0.95)	0.85 (0.74, 0.99)	0.73 (0.58, 0.93)	0.89 (0.82, 0.96)
Model 2, + season of blood draw	19,866	1.0 (ref)	0.81 (0.68, 0.97)	0.81 (0.69, 0.95)	0.83 (0.70, 0.98)	0.72 (0.55, 0.95)	0.88 (0.80, 0.97)
Model 2, + family history and disease prevalence‡	17,183	1.0 (ref)	0.82 (0.65, 1.03)	0.79 (0.65, 0.96)	0.84 (0.65, 1.08)	0.74 (0.53, 1.04)	0.90 (0.82, 0.98)
Model 2, + hormone use and menopause status in women	19,907	1.0 (ref)	0.81 (0.68, 0.96)	0.81 (0.70, 0.94)	0.83 (0.72, 0.96)	0.71 (0.55, 0.91)	0.88 (0.80, 0.96)
Model 2, + BMI-GRS and IR-GRS§	16162	1.0 (ref)	0.84 (0.69, 1.03)	0.81 (0.7, 0.95)	0.82 (0.7, 0.96)	0.74 (0.59, 0.94)	0.88 (0.82, 0.96)
Model 2, + diet quality score§	19909	1.0 (ref)	0.81 (0.69, 0.97)	0.81 (0.69, 0.96)	0.84 (0.72, 0.97)	0.74 (0.57, 0.95)	0.89 (0.82, 0.97)
Plasma composite biomarker score							
Model 2†	18,276	1.0 (ref)	0.77 (0.68, 0.87)	0.66 (0.54, 0.80)	0.59 (0.48, 0.72)	0.50 (0.40, 0.62)	0.75 (0.67, 0.83)
Model 2, with multiple imputation	22,742	1.0 (ref)	0.78 (0.70, 0.87)	0.65 (0.53, 0.79)	0.57 (0.48, 0.69)	0.50 (0.41, 0.63)	0.75 (0.68, 0.83)
Model 2, excluding HbA1c ≥ 6.5%	16,840	1.0 (ref)	0.82 (0.73, 0.92)	0.70 (0.57, 0.86)	0.62 (0.50, 0.76)	0.56 (0.44, 0.70)	0.78 (0.70, 0.86)
Model 2, excluding first 2 years' incident T2D	17,572	1.0 (ref)	0.80 (0.71, 0.89)	0.68 (0.56, 0.83)	0.61 (0.50, 0.75)	0.53 (0.43, 0.65)	0.77 (0.70, 0.84)
Model 2, excluding first 4 years' incident T2D	16,542	1.0 (ref)	0.84 (0.74, 0.96)	0.72 (0.58, 0.88)	0.64 (0.51, 0.80)	0.54 (0.42, 0.70)	0.78 (0.70, 0.86)
Model 2, restricting to first 8 years' follow up	18,279	1.0 (ref)	0.77 (0.68, 0.87)	0.66 (0.54, 0.80)	0.59 (0.49, 0.71)	0.50 (0.40, 0.62)	0.74 (0.66, 0.83)
Model 2, excluding baseline cancer and CVD cases	17,386	1.0 (ref)	0.75 (0.66, 0.86)	0.64 (0.53, 0.78)	0.57 (0.46, 0.70)	0.48 (0.38, 0.60)	0.74 (0.66, 0.83)
NA							
Model 2, + vitamin supplements	16,699	1.0 (ref)	0.75 (0.65, 0.86)	0.68 (0.57, 0.81)	0.60 (0.49, 0.73)	0.50 (0.40, 0.64)	0.76 (0.68, 0.85)
Model 2, + season of blood draw	18,235	1.0 (ref)	0.75 (0.66, 0.86)	0.64 (0.52, 0.79)	0.57 (0.46, 0.71)	0.48 (0.38, 0.62)	0.74 (0.66, 0.83)
Model 2, + family history and disease prevalence‡	15,609	1.0 (ref)	0.78 (0.69, 0.88)	0.71 (0.59, 0.86)	0.65 (0.53, 0.79)	0.55 (0.46, 0.65)	0.78 (0.70, 0.87)
Model 2, + hormone use and menopause status in women	18,276	1.0 (ref)	0.77 (0.68, 0.88)	0.65 (0.53, 0.80)	0.58 (0.47, 0.72)	0.49 (0.39, 0.62)	0.74 (0.66, 0.83)
Model 2, + BMI-GRS and IR-GRS§	14722	1.0 (ref)	0.79 (0.66, 0.96)	0.66 (0.56, 0.78)	0.58 (0.49, 0.68)	0.5 (0.41, 0.62)	0.76 (0.71, 0.81)
Model 2, + diet quality score§	18279	1.0 (ref)	0.77 (0.68, 0.87)	0.66 (0.54, 0.81)	0.59 (0.48, 0.72)	0.5 (0.40, 0.62)	0.75 (0.68, 0.83)

* Hazard Ratios (HRs) at each category Q2-Q5 (compared with Q1) and per 1-standard deviation (SD) of each biomarker, estimated from country-specific Prentice-weighted Cox regression models; estimates were then combined across countries using random-effects meta-analysis; SD was 19.2 µmol/L for vitamin C, 0.83 µmol/L for total carotenoids, 0.12 µmol/L for α-carotene, 0.41 µmol/L for β-carotene, 0.27µmol/L for lycopene, 0.15 µmol/L for lutein, 0.04 µmol/L for zeaxanthin, 0.29 µmol/L for β-cryptoxanthin and 0.57 for composite biomarker score;

† Model 2: the same as model 2 in the main analysis, with adjustment for age (as underlying timescale), sex, centre, physical activity, smoking status, employment, marital status, education, alcohol intake, total energy intake, HDL-C (not for vitamin C), LDL-C (not for vitamin C), BMI and waist circumference;

‡ Baseline prevalence of stroke, heart diseases and any cancer and family history of diabetes.

§ BMI-GRS and IR-GRS refer to unweighted genetic risk score of body-mass index and insulin resistance, respectively. Diet quality score refers to the adherence to the Mediterranean diet score (low [score 0-6], middle [score 7-10], and high [score 11-18]).

Supplemental Table S4 Sensitivity analysis for the influence of individual biomarkers on the results of the composite biomarker score

Composite biomarker score	n	Hazard ratios (95% Confidence Intervals)*
including all 7 biomarkers	18279	0.75 (0.67, 0.83)
excluding vitamin C	19909	0.77 (0.70, 0.85)
excluding lutein	18279	0.76 (0.69, 0.84)
excluding zeaxanthin	18279	0.73 (0.66, 0.81)
excluding β-cryptoxanthin	18279	0.76 (0.69, 0.84)
excluding α-carotene	18279	0.76 (0.68, 0.85)
excluding β-carotene	18279	0.78 (0.70, 0.86)
excluding lycopene	18279	0.75 (0.68, 0.83)

* Hazard Ratios (HRs) per 1-standard deviation (SD) of the composite biomarker score, estimated from country-specific Prentice-weighted Cox regression models; estimates were then combined across countries using random-effects meta-analysis. The analyses were based on model 2 in the main analysis with adjustment for age (as underlying timescale), sex, centre, physical activity, smoking status, employment, marital status, education, alcohol intake, total energy intake, HDL-C (not for vitamin C), LDL-C (not for vitamin C), BMI and waist circumference.

Supplemental Figure S1 EPIC-InterAct study design and participants included in the analysis

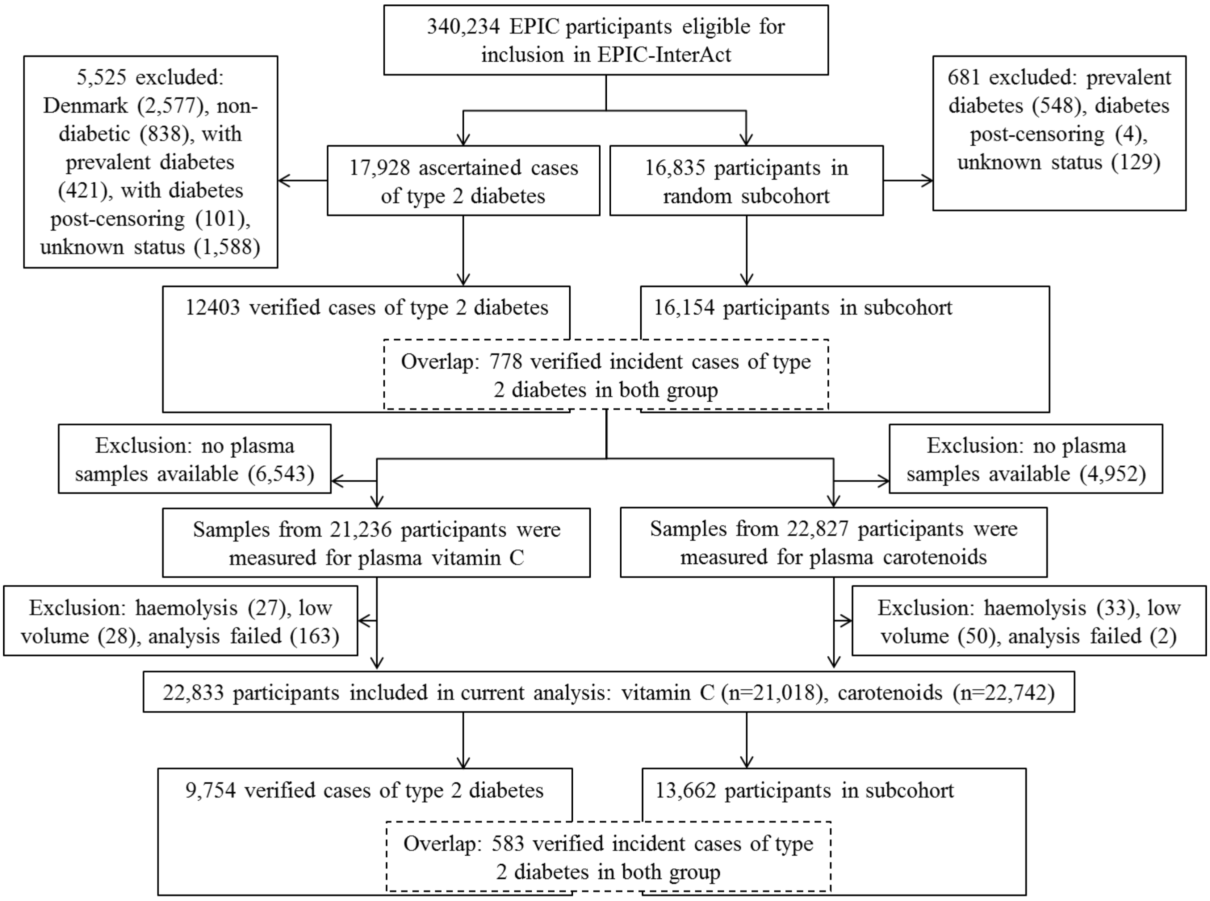

Supplemental Figure S2 Summary of the association of demographic and lifestyle factors with plasma vitmain C and carotenoids in the subcohort of EPIC-InterAct study. The values in the box represent the differences in vitamin C and total carotenoids (in SD unit) per 1-standardised unit/category difference in each demographic or lifestyle factors. The mean (SD) or % of participants for the examined demographic, lifestyle factors were presented in the second column. Linear regression was used to obtain the country-specific estimate of an association with mutual adjustment for all the other examined demographic, lifestyle factors if available in that country. The country-specific estimates were then combined using random effects meta-analysis. *indicates p<0.05, ** indicates p<0.001, *** indicates p<0.0001. Sample size was 10,584 for vitamin C and 11,537 for the carotenoids estimation. All the values are expressed in red scale for different levels of positive associations and blue scale for different levels of negative associations. All of the carotenoids variables were natural log-transformed before statistical analysis in the linear regression. SD was 19.2 µmol/L for vitamin C, 0.50 for total carotenoids (ln-transformed), 0.91 for α-carotene (ln-transformed), 0.73 for β-carotene (ln-transformed), 0.68 for lycopene (ln-transformed), 0.51 for lutein (ln-transformed), 0.91 for zeaxanthin (ln-transformed), 0.94 for β-cryptoxanthin (ln-transformed) and 0.57 for CB-score (composite biomarker score).

	Mean (SD)/ %	Vitamin C	Total carotenoids	α-carotene	β-carotene	Lycopene	Lutein	Zeaxanthin	β-cryptoxanthin	CB-score
Sex (ref: men), women	62.6	0.22 ***	0.07 *	0.09 *	0.14 *	-0.10 *	0	-0.06 *	0.15 ***	0.10 *
Age (per 5 y)	52 (9)	0.01 *	0.01	0.02	0.05 ***	-0.10***	0.07 ***	0	0.02	0.02
BMI (per 5 kg/m²)	26 (4)	0.02	-0.09 *	-0.05 *	-0.08 *	-0.03	-0.13***	-0.04 *	-0.02	-0.08***
Waist circumference (per 5 cm)	87 (13)	-0.06***	-0.07 ***	-0.07***	-0.09***	-0.03 *	-0.03 **	-0.01	-0.05 ***	-0.06***
Alcohol (per 15 g/d)	13 (19)	0	-0.05 ***	-0.05***	-0.10***	0.03	0	-0.02	-0.05 ***	-0.02 *
Season of blood draw (ref: winter), spring	27.8	-0.03	-0.05	-0.07 *	0	0.08 *	0.13 ***	0.17 ***	-0.28 ***	-0.02
Season of blood draw (ref: winter), summer	19.9	0.01	0.02	0.03	0.18 ***	0.29 ***	0.12 *	0.32 ***	-0.45 ***	0.05
Season of blood draw (ref: winter), autumn	27.9	-0.05	-0.01	0.13 ***	0.17 ***	0.21 ***	0.04	0.05	-0.49 ***	-0.02
Smoking status (ref: never), former	26.5	-0.02	-0.05 *	-0.04 *	-0.07 **	0.01	-0.03	0.01	-0.07 ***	-0.04
Smoking status (ref: never), current	25.7	-0.17***	-0.28 ***	-0.31***	-0.26***	-0.06 *	-0.22***	-0.08 *	-0.34 ***	-0.27***
Physical activity (ref: inactive), moderate inactive	33.0	0.06 *	0.03	0.02	0	0.05 *	0.01	0.02	0.03	0.04
Physical activity (ref: inactive), moderate active	22.5	0.09 *	0.08 **	0.08 *	0.09 **	0.06 *	0.07 *	0.01	0.04	0.09***
Physical activity (ref: inactive), active	20.5	0.13 *	0.11 ***	0.07 *	0.09 **	0.07 *	0.10 **	0.06 *	0.09 **	0.15***
Education level (ref: low), middle	38.6	0	0.06	0.09 *	0.04	0.09	0.05	0.03	0.01	0.04
Education level (ref: low), high	20.4	0.08 *	0.15 *	0.21 ***	0.16 **	0.12	0.10 *	0.04	0.08 **	0.13 *
Employment (ref: no employment), yes	66.8	0.03	0.08 **	0.05 *	0.04	0.09 **	0.07 *	0.04	0.10 ***	0.06 *
Marital status (ref: single), married	79.5	-0.06	0.02	0.01	0.03	0.08	0.03	0.03	0.03	-0.04
Marital status (ref: single), separated/divorced	7.2	-0.05	-0.01	-0.04	-0.04	0.06	-0.08	0.04	0.02	-0.08
Marital status (ref: single), widowed	4.0	-0.21 *	0.03	0	-0.03	0.15	-0.08	0.01	0.04	-0.15 *

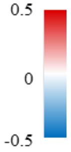

Supplemental Figure S3 Association of dietary fruit and vegetable with plasma vitmain C and carotenoids stratified by country in the subcohort of EPIC-InterAct study. The x-axis values in the figure represent standardised difference (error bar: 95%CI) in vitamin C and carotenoids (in SD unit) per 100 g/d difference in each fruit and vegetable subgroup. Linear regression was used to obtain the country-specific estimate of an association with mutual adjustment for all the other examined demographic, lifestyle or dietary factors if available in that country. The country-specific estimates were then combined using random effects meta-analysis. All of the carotenoids variables were natural log-transformed before statistical analysis in the linear regression. SD was 19.2 µmol/L for vitamin C, 0.50 for total carotenoids (ln-transformed), 0.91 for α-carotene (ln-transformed), 0.73 for β-carotene (ln-transformed), 0.68 for lycopene (ln-transformed), 0.51 for lutein (ln-transformed), 0.91 for zeaxanthin (ln-transformed), 0.94 for β-cryptoxanthin (ln-transformed) and 0.57 for composite biomarker score. Citrus fruits included grapefruit, orange, tangerine, lemon; Non-citrus fruits included apple, pear, grape, apricot, cherry, peach, plum, nectarine, prune, melon, pineapple, strawberry, raspberry, banana, kiwi; green leafy vegetables included spinach, chard, endive, lettuce, borage, watercress, beet leaves; fruiting vegetables included tomato, pepper, avocado, courgette, artichoke, aubergine, pumpkin, squash; root vegetables included carrot, radish, salsify, beetroot, turnip, celeriac, swede; cabbage included broccoli, cabbage, brussel sprouts, cauliflower, kale; other vegetables: vegetables not included in the above groups.

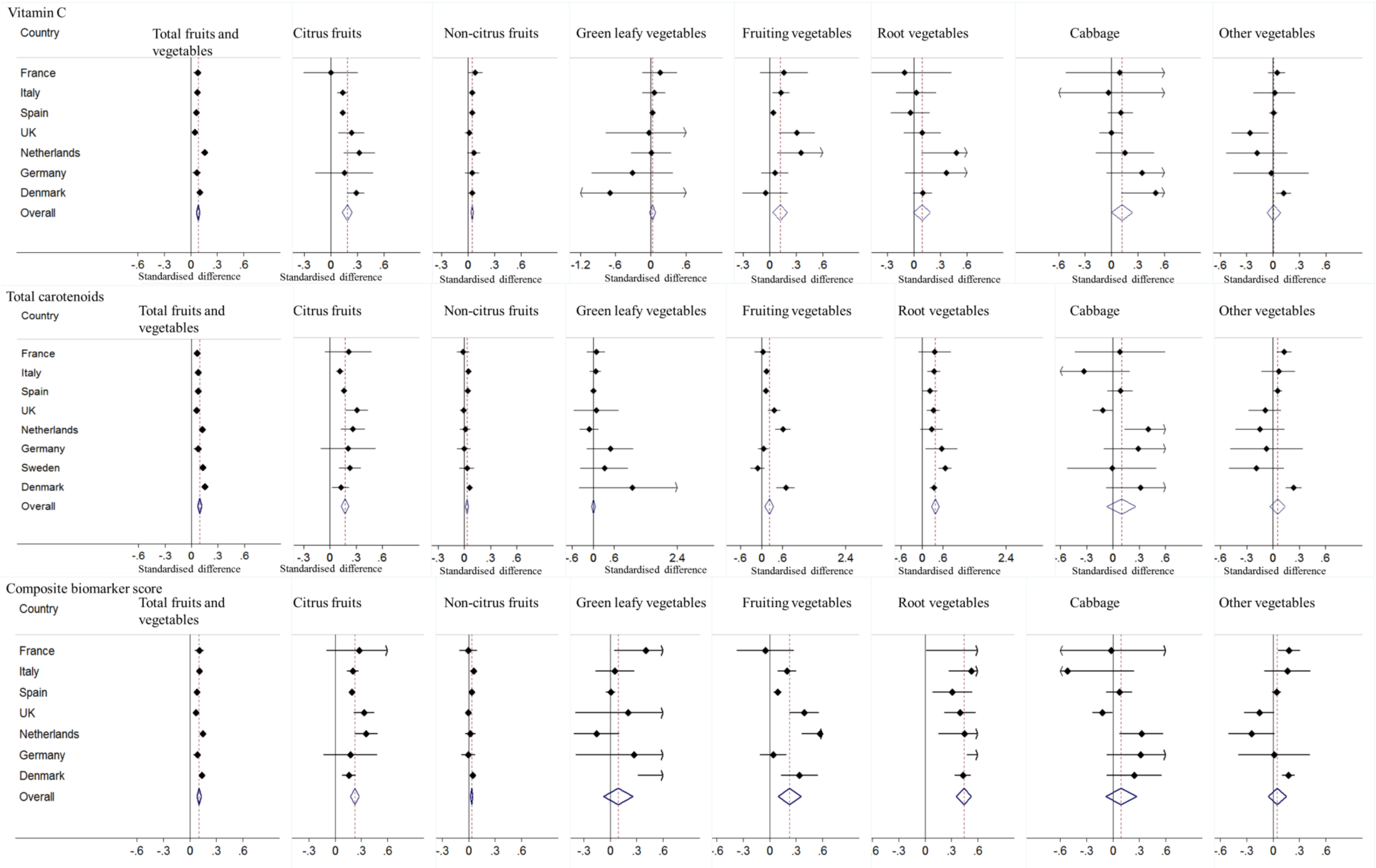
Vitamin C
Country
Total fruits and vegetables
Citrus fruits
Non-citrus fruits
Green leafy vegetables
Fruiting vegetables
Root vegetables
Cabbage
Other vegetables
France
Italy
Spain
UK
Netherlands
Germany
Denmark
Overall
Standardised difference
Total carotenoids
Sweden
Composite biomarker score

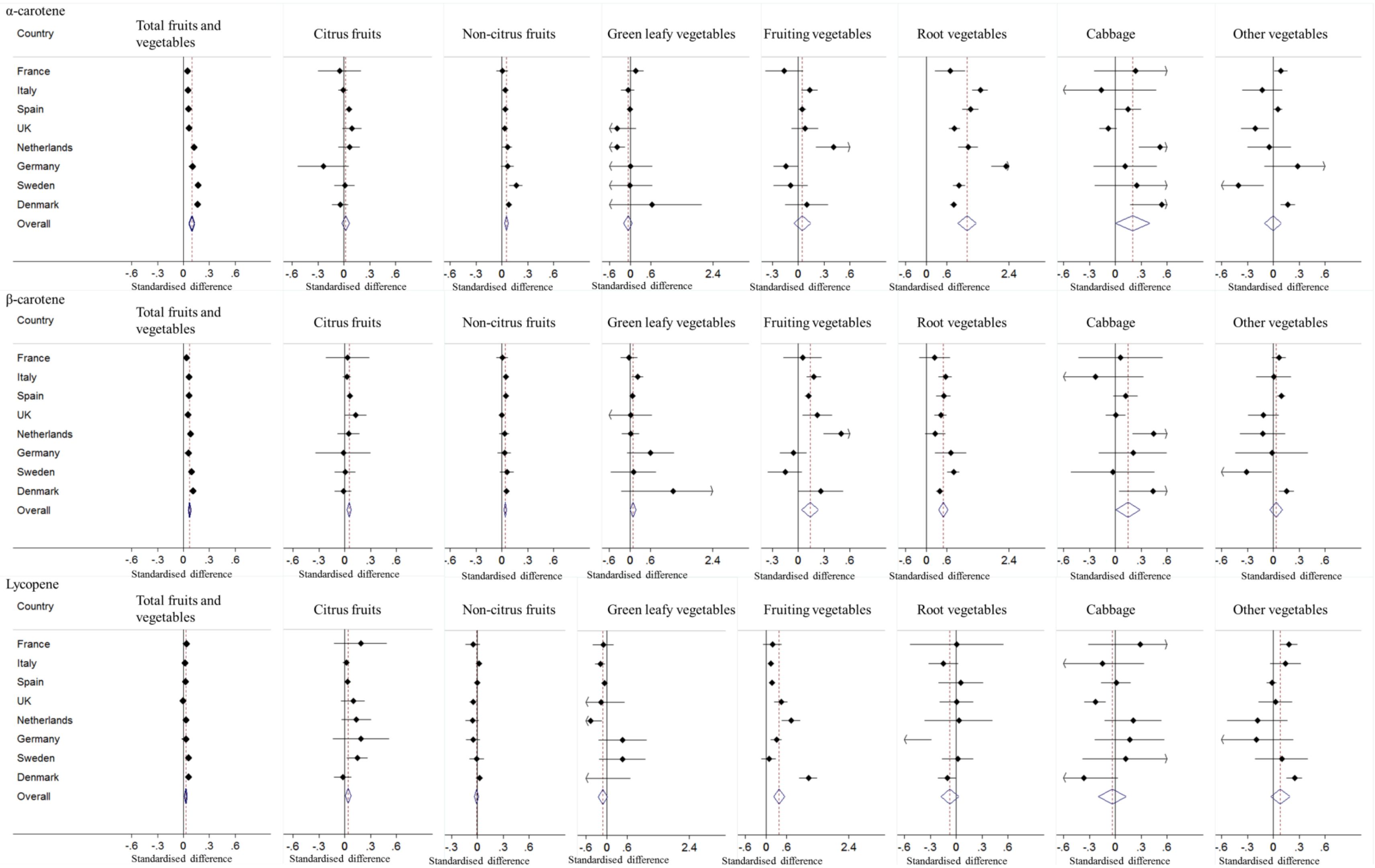
α-carotene
β-carotene
Lycopene
Country
Total fruits and vegetables
Citrus fruits
Non-citrus fruits
Green leafy vegetables
Fruiting vegetables
Root vegetables
Cabbage
Other vegetables
France
Italy
Spain
UK
Netherlands
Germany
Sweden
Denmark
Overall
Standardised difference

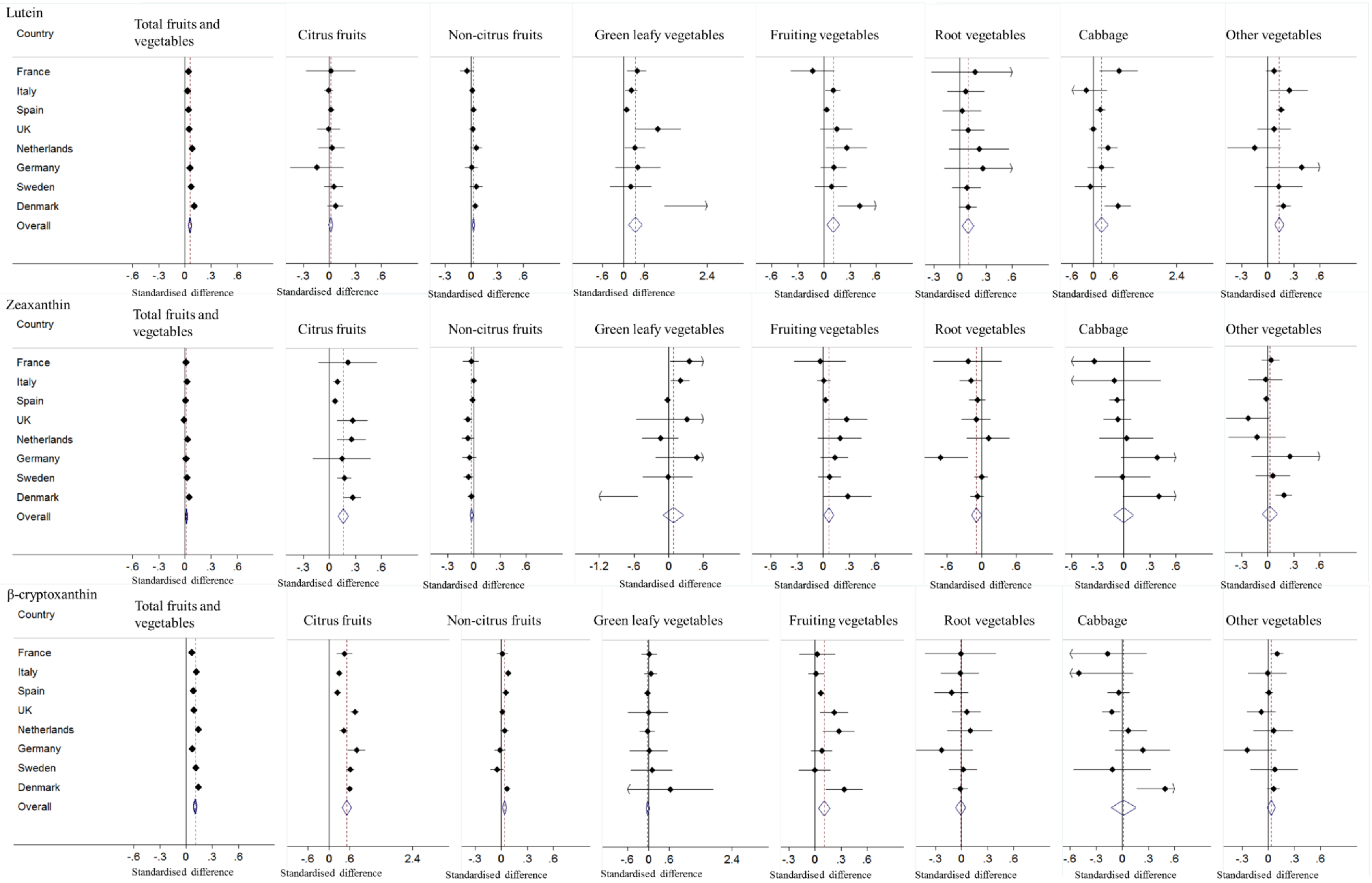
Lutein
Country
Total fruits and vegetables
Citrus fruits
Non-citrus fruits
Green leafy vegetables
Fruiting vegetables
Root vegetables
Cabbage
Other vegetables
France
Italy
Spain
UK
Netherlands
Germany
Sweden
Denmark
Overall
Standardised difference
Zeaxanthin
β-cryptoxanthin

Supplemental Figure S4 Association of plasma vitamin C and carotenoids (per 1-standard deviation) with type 2 diabetes by countries in the EPIC-InterAct study. Hazard Ratios (HRs) are per 1-standard deviation (SD) of each biomarker, estimated from country-specific Prentice-weighted Cox regression models, with adjustment for age (as underlying timescale), sex, centre, physical activity, smoking status, employment, marital status, education, alcohol intake, total energy intake, HDL-C (not for vitamin C), LDL-C (not for vitamin C), BMI and waist circumference; estimates were then combined across countries using random-effects meta-analysis. SD was 19.2 µmol/L for vitamin C, 0.83 µmol/L for total carotenoids, 0.12 µmol/L for α-carotene, 0.41 µmol/L for β-carotene, 0.27µmol/L for lycopene, 0.15 µmol/L for lutein, 0.04 µmol/L for zeaxanthin, 0.29 µmol/L for β-cryptoxanthin, and 0.57 for composite biomarker score.

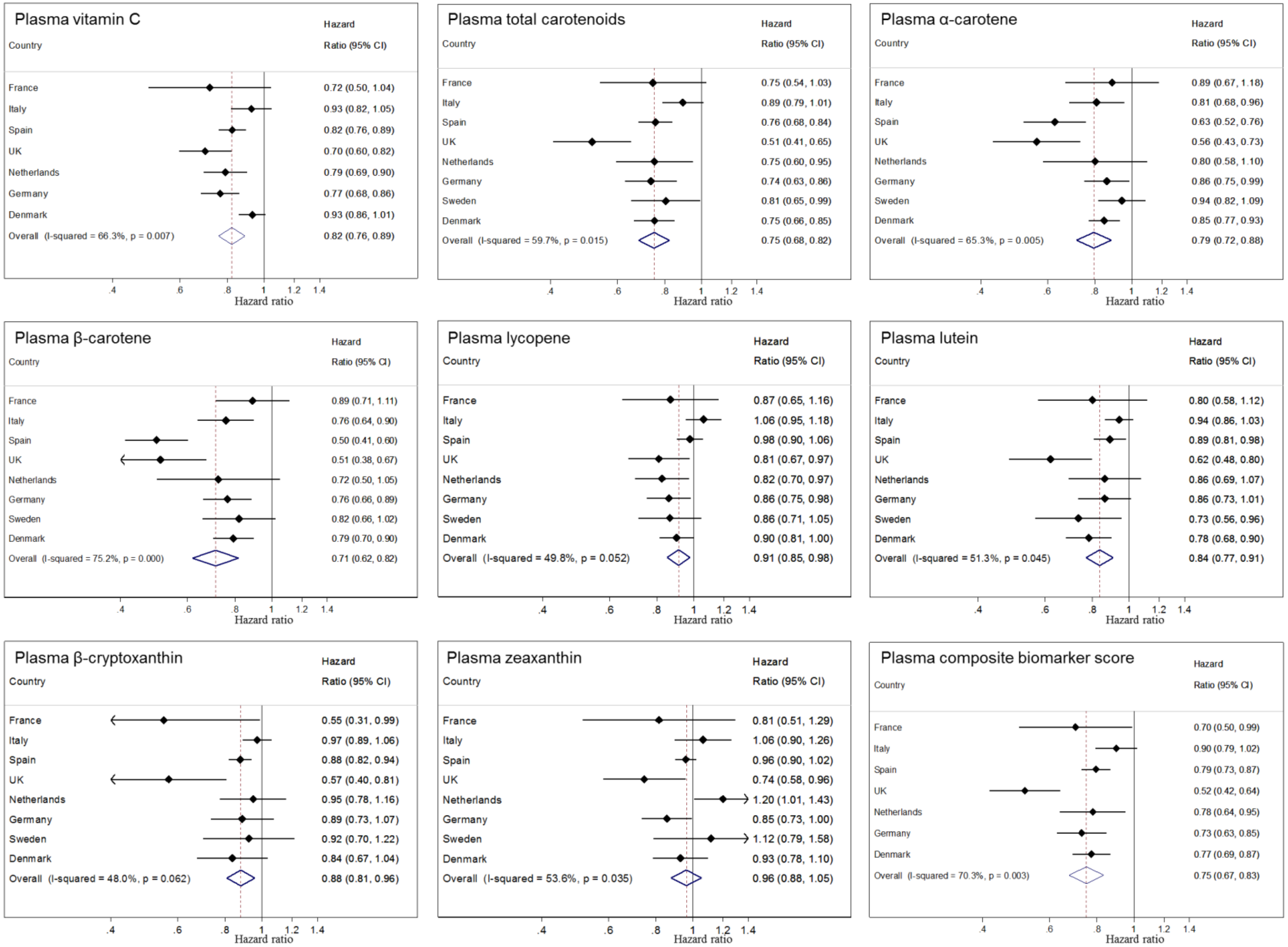
Plasma vitamin C
Country
Hazard Ratio (95% CI)
France 0.72 (0.50, 1.04)
Italy 0.93 (0.82, 1.05)
Spain 0.82 (0.76, 0.89)
UK 0.70 (0.60, 0.82)
Netherlands 0.79 (0.69, 0.90)
Germany 0.77 (0.68, 0.86)
Denmark 0.93 (0.86, 1.01)
Overall (I-squared = 66.3%, p = 0.007) 0.82 (0.76, 0.89)
.4 .6 .8 1 1.2 1.4
Hazard ratio
Plasma total carotenoids
Country
Hazard Ratio (95% CI)
France 0.75 (0.54, 1.03)
Italy 0.89 (0.79, 1.01)
Spain 0.76 (0.68, 0.84)
UK 0.51 (0.41, 0.65)
Netherlands 0.75 (0.60, 0.95)
Germany 0.74 (0.63, 0.86)
Sweden 0.81 (0.65, 0.99)
Denmark 0.75 (0.66, 0.85)
Overall (I-squared = 59.7%, p = 0.015) 0.75 (0.68, 0.82)
.4 .6 .8 1 1.2 1.4
Hazard ratio
Plasma α-carotene
Country
Hazard Ratio (95% CI)
France 0.89 (0.67, 1.18)
Italy 0.81 (0.68, 0.96)
Spain 0.63 (0.52, 0.76)
UK 0.56 (0.43, 0.73)
Netherlands 0.80 (0.58, 1.10)
Germany 0.86 (0.75, 0.99)
Sweden 0.94 (0.82, 1.09)
Denmark 0.85 (0.77, 0.93)
Overall (I-squared = 65.3%, p = 0.005) 0.79 (0.72, 0.88)
.4 .6 .8 1 1.2 1.4
Hazard ratio
Plasma β-carotene
Country
Hazard Ratio (95% CI)
France 0.89 (0.71, 1.11)
Italy 0.76 (0.64, 0.90)
Spain 0.50 (0.41, 0.60)
UK 0.51 (0.38, 0.67)
Netherlands 0.72 (0.50, 1.05)
Germany 0.76 (0.66, 0.89)
Sweden 0.82 (0.66, 1.02)
Denmark 0.79 (0.70, 0.90)
Overall (I-squared = 75.2%, p = 0.000) 0.71 (0.62, 0.82)
.4 .6 .8 1 1.2 1.4
Hazard ratio
Plasma lycopene
Country
Hazard Ratio (95% CI)
France 0.87 (0.65, 1.16)
Italy 1.06 (0.95, 1.18)
Spain 0.98 (0.90, 1.06)
UK 0.81 (0.67, 0.97)
Netherlands 0.82 (0.70, 0.97)
Germany 0.86 (0.75, 0.98)
Sweden 0.86 (0.71, 1.05)
Denmark 0.90 (0.81, 1.00)
Overall (I-squared = 49.8%, p = 0.052) 0.91 (0.85, 0.98)
.4 .6 .8 1 1.2 1.4
Hazard ratio
Plasma lutein
Country
Hazard Ratio (95% CI)
France 0.80 (0.58, 1.12)
Italy 0.94 (0.86, 1.03)
Spain 0.89 (0.81, 0.98)
UK 0.62 (0.48, 0.80)
Netherlands 0.86 (0.69, 1.07)
Germany 0.86 (0.73, 1.01)
Sweden 0.73 (0.56, 0.96)
Denmark 0.78 (0.68, 0.90)
Overall (I-squared = 51.3%, p = 0.045) 0.84 (0.77, 0.91)
.4 .6 .8 1 1.2 1.4
Hazard ratio
Plasma β-cryptoxanthin
Country
Hazard Ratio (95% CI)
France 0.55 (0.31, 0.99)
Italy 0.97 (0.89, 1.06)
Spain 0.88 (0.82, 0.94)
UK 0.57 (0.40, 0.81)
Netherlands 0.95 (0.78, 1.16)
Germany 0.89 (0.73, 1.07)
Sweden 0.92 (0.70, 1.22)
Denmark 0.84 (0.67, 1.04)
Overall (I-squared = 48.0%, p = 0.062) 0.88 (0.81, 0.96)
.4 .6 .8 1 1.2 1.4
Hazard ratio
Plasma zeaxanthin
Country
Hazard Ratio (95% CI)
France 0.81 (0.51, 1.29)
Italy 1.06 (0.90, 1.26)
Spain 0.96 (0.90, 1.02)
UK 0.74 (0.58, 0.96)
Netherlands 1.20 (1.01, 1.43)
Germany 0.85 (0.73, 1.00)
Sweden 1.12 (0.79, 1.58)
Denmark 0.93 (0.78, 1.10)
Overall (I-squared = 53.6%, p = 0.035) 0.96 (0.88, 1.05)
.4 .6 .8 1 1.2 1.4
Hazard ratio
Plasma composite biomarker score
Country
Hazard Ratio (95% CI)
France 0.70 (0.50, 0.99)
Italy 0.90 (0.79, 1.02)
Spain 0.79 (0.73, 0.87)
UK 0.52 (0.42, 0.64)
Netherlands 0.78 (0.64, 0.95)
Germany 0.73 (0.63, 0.85)
Denmark 0.77 (0.69, 0.87)
Overall (I-squared = 70.3%, p = 0.003) 0.75 (0.67, 0.83)
.4 .6 .8 1 1.2 1.4
Hazard ratio

Supplemental Figure S5 Association between plasma vitamin C, carotenoids and type 2 diabetes (per 1-standard deviation) stratified by baseline age, BMI and vitamin supplement subgroups in the EPIC-InterAct study. Interaction of plasma vitamin C and carotenoids with baseline age, sex, BMI, physical activity, smoking status, season and vitamin supplements for type 2 diabetes was examined and the interactions with p-value<0.05 were presented. * indicated that the p-value for the interaction analysis passed the Bonferroni correction (p-interaction threshold for the multiple correction= 0.0008). Values were presented as hazard ratio (95%CI) of type 2 diabetes per 1-standard deviation (SD) difference in each biomarker stratified by the age, BMI or vitamin supplement subgroup. The statistical model in the stratified analysis was the same as the model 2 except that the samples were divided into several subgroups, adjusted for age (as underlying timescale), sex, centre, physical activity, smoking status, employment, marital status, education, alcohol intake, total energy intake, HDL-C (not for vitamin C), LDL-C (not for vitamin C), BMI and waist circumference. SD was 19.2 µmol/L for vitamin C, 0.83 µmol/L for total carotenoids, 0.12 µmol/L for α-carotene, 0.41 µmol/L for β-carotene, 0.27µmol/L for lycopene, 0.15 µmol/L for lutein, 0.04 µmol/L for zeaxanthin, 0.29 µmol/L for β-cryptoxanthin, and 0.57 for composite biomarker score.

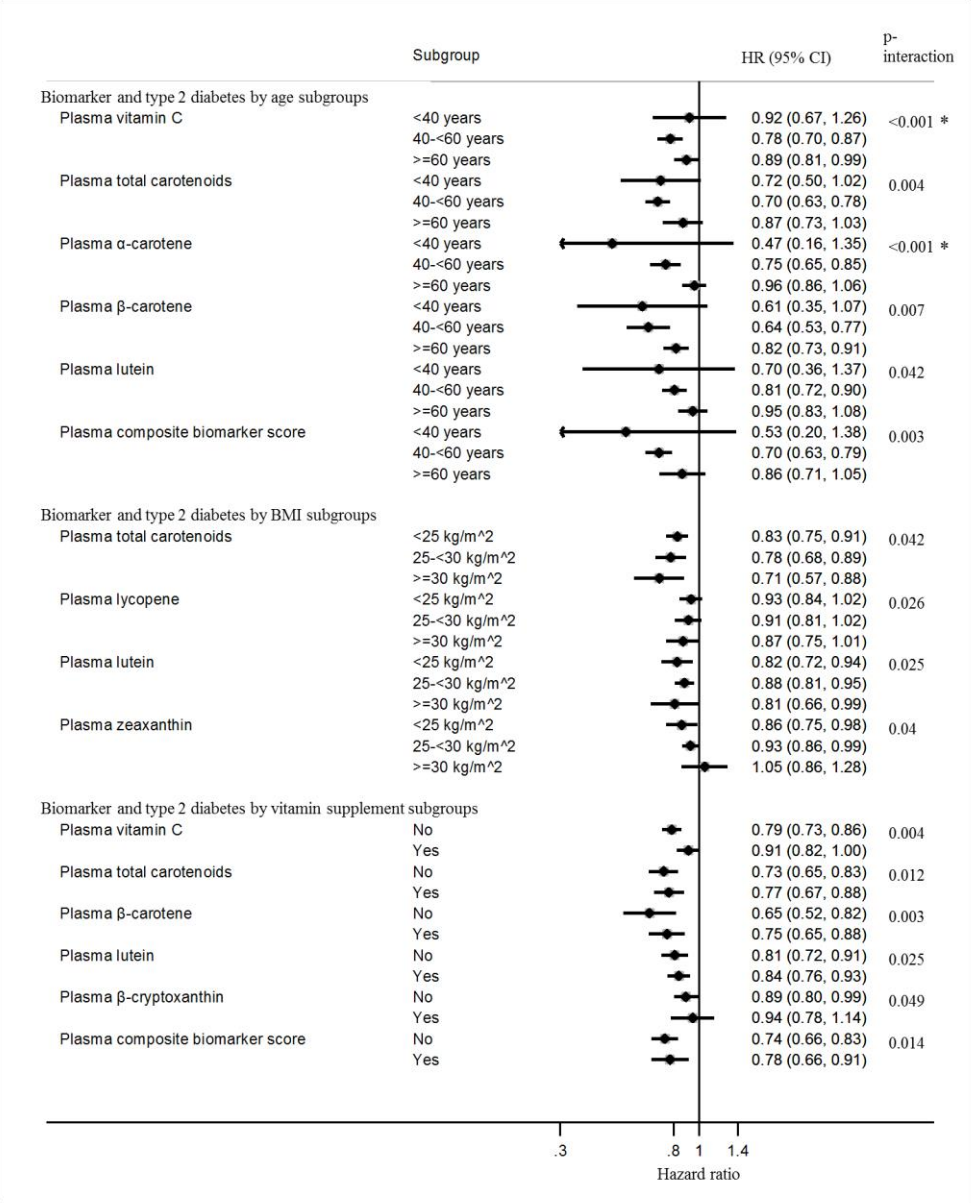

Supplement: Supplementary file 1 — Web appendix: Online supplemental files [file zhej053745.ww.pdf]
